# Vaccinia-related kinase 2 variants differentially affect breast cancer growth by regulating kinase activity

**DOI:** 10.32604/or.2023.031031

**Published:** 2023-12-28

**Authors:** SEUNG-HEE GWAK, JUHYUN LEE, EUNJI OH, DOHYUN LEE, WONSHIK HAN, JONGMIN KIM, KYONG-TAI KIM

**Affiliations:** 1 Department of Life Sciences, Pohang University of Science and Technology, Pohang, 37673, Korea; 2 R&D Center, NovMetaPharma Co., Ltd., Pohang, 37668, Korea; 3 Department of Surgery and Cancer Research Institute, Seoul National University College of Medicine, Seoul, 03080, Korea; 4 Generative Genomics Research Center, Global Green Research & Development Center, Handong Global University, Pohang, 37554, Korea

**Keywords:** VRK2, Kinase activity, Breast cancer, Tumor, RNA editing, Cell proliferation, Cell growth

## Abstract

Genetic information is transcribed from genomic DNA to mRNA, which is then translated into three-dimensional proteins. mRNAs can undergo various post-transcriptional modifications, including RNA editing that alters mRNA sequences, ultimately affecting protein function. In this study, RNA editing was identified at the 499th base (c.499) of human vaccinia-related kinase 2 (VRK2). This RNA editing changes the amino acid in the catalytic domain of VRK2 from isoleucine (with adenine base) to valine (with guanine base). Isoleucine-containing VRK2 has higher kinase activity than the valine-containing VRK2, which leads to an increase in tumor cell proliferation. Earlier we reported that VRK2 directly interacts with dystrobrevin-binding protein (dysbindin) and results in reducing its stability. Herein, we demonstrate that isoleucine-containing VRK2 decreases the level of dysbindin than valine-containing VRK2. Dysbindin interacts with cyclin D and thereby regulates its expression and function. The reduction in the level of dysbindin by isoleucine-containing VRK2 further enhances the cyclin D expression, resulting in increased tumor growth and reduction in survival rates. It has also been observed that in patient samples, VRK2 level was elevated in breast cancer tissue compared to normal breast tissue. Additionally, the isoleucine form of VRK2 exhibited a greater increase in breast cancer tissue. Therefore, it is concluded that VRK2, especially dependent on the 167th variant amino acid, can be one of the indexes of tumor progression and proliferation.

## Introduction

Cancer causes the most deaths worldwide. The incidence of cancer and cancer deaths continue to increase over time, and in 2020, approximately 10 million cancer-related deaths have occurred [1]. Among cancer types, breast cancer is the most common cancer found in women and causes their deaths [2]. Many studies have revealed that breast cancer is caused by environmental factors, such as obesity [3], heavy smoking [4], and genetic factors such as breast cancer gene *(*BRCA)*1*, *BRCA2*, phosphatase and tensin homolog (*PTEN*), tumor protein P53 (*TP53*), cadherin 1 (*CDH1*), and serine/threonine kinase 11 (*STK11*) [5].

Recently, several studies have shown how vaccinia-related kinase 2 (VRK2) is related to cancer. VRK2 is an active serine/threonine kinase and the second member of the VRK family. Thus far, very little is known about the substrates and functional roles of VRK2. VRK2 phosphorylates ubiquitin-specific protease 25 (USP25) and inhibits the deubiquitinating activity of USP25, which further increases polyglutamine aggregation [6,7]. In addition, VRK2 phosphorylates the nuclear factor of activated T cells 1 (NFAT1) and modulates breast cancer cell invasion through elevated expression of cyclooxygenase 2 (COX2) [8]. Moreover, VRK2 phosphorylates inhibitor of NF-κB kinase (IKKβ) and activates tumor necrosis factor-α/nuclear factor-kappa signaling, which leads to pancreatic cancer progression [9]. However, the mechanism behind the role of VRK2 in tumor pathology remains unknown.

In this study, VRK2 was found to have two types of amino acid variants distinguished by ribonucleic acid (RNA) editing. Previous studies have shown that RNA editing is related to the pathology of cancer [10]. RNA editing occurs when the RNA sequences are changed without concomitant changes to the deoxyribonucleic acid (DNA) sequences. In RNA editing, base substitution occurs through the deamination of adenosine or cytosine. The most abundant form of RNA editing is adenosine-to-inosine (A-to-I) substitution, which is achieved by the deamination of A through adenosine deaminase acting on RNA (ADAR) enzymes. As I is treated as guanine (G) in most cellular processes, A-to-I RNA editing is often called A-to-G RNA editing [11,12]. Another type of RNA editing is G-to-A substitution, which was reported to happen in Wilms tumor 1 (WT1), tryptophan hydroxylase 2 (TPH2), and heterogeneous nuclear ribonucleoprotein K (hnRNP K) [13–15]. However, very little is known about its chemical mechanism or mediating enzyme, whereas apolipoprotein B mRNA editing enzyme catalytic subunit 3A (Apobec3A) is the only known enzyme involved in G-to-A RNA editing [13]. RNA editing can occur in coding sequences, which results in the alteration of amino acid sequence, leading to changes in the conformation and function of proteins [16]. Several reports have indicated that functional changes in proteins due to RNA editing can affect cancer pathogenesis. For example, A-to-G RNA editing of Antizyme Inhibitor 1 (AZIN1), which leads to S367G amino acid substitution, causes a conformational change to the AZIN1 protein. Conformationally changed AZIN1 protein has a stronger affinity to the antizyme, promoting cell proliferation [16]. A-to-G editing of Rho-related GTP-binding protein (RhoQ), which leads to N136S amino acid substitution, increases RhoQ activity and invasion by colorectal cancer [17].

In this study, we found RNA editing of VRK2 at the 499th base (c.499). The amino acid 167 changes through RNA editing, and this amino acid substitution can affect VRK2 kinase activity. Amino acid variation of VRK2 affects tumor growth by regulating dysbindin and cyclin D in mouse and human breast cancer tissue.

## Materials and Methods

### Genomic DNA (gDNA) preparation

Harvested cells or tissues were resuspended in digestion buffer (100 mM NaCl, 10 mM Tris pH 8.0, 25 mM ethylenediaminetetraacetic acid [EDTA], 0.5% sodium dodecyl sulfate [SDS], and 0.1 mg/mL proteinase K). Resuspended cells were incubated for 12–18 h at 50°C. Then, 1 volume of phenol/chloroform/isoamyl alcohol (c9017, Bioneer, Daejeon, South Korea) was added, and samples were centrifuged at 15,000 rpm for 20 min. The aqueous layer of the sample was transferred into a new tube. Thereafter, 1/10 volume of 3 M NaOAc (pH 5.2) and 2 volumes of 100% EtOH were added and mixed by vortexing. Samples were centrifuged at 15,000 rpm for 3 min. The supernatant was discarded, and the gDNA pellet was resuspended in distilled water.

### cDNA preparation

Total RNA was extracted from cell lines and human tissues using a TRI solution (TS200-001, Bioscience Technology, NJ, USA) according to the manufacturer’s instructions. The cDNA was prepared by reverse transcription of mRNA. Reverse transcription reactions were catalyzed by Improm-II reverse transcriptase (A3803, Promega, WI, USA).

### Sequencing

The prepared gDNA and cDNA were sequenced using a commercial sequencing service offered by Macrogen, South Korea. Polymerase chain reaction (PCR) analyses were performed to amplify human VRK2, and sequencing service was requested using the following sequencing primers: TGTCCCATTATGGCCTTTTC for cDNA and AGGATGAGCAACCAGAAGATG for gDNA.

### In vitro kinase assay and immunoprecipitation

Kinase assays were performed with 1, 1.5, and 2 μg of 167V VRK2 and 2 μg of 167I VRK2. The VRK2 forms were expressed in *Escherichia coli* using the pTYB2 vector (intein tag) and purified by pull-down assays using the chitin bead system. The reaction was performed at 37°C in a kinase buffer solution [20 mM Tris-HCl pH 7.5, 5 mM MgCl_2_, 0.5 mM dithiothreitol, 150 mM KCl, and (γ-^32^P)ATP]. The reaction was stopped after 1 h, and proteins were separated by SDS polyacrylamide gel electrophoresis, and visualized with autoradiography.

The immunoprecipitated VRK2 was obtained from flag-tagged VRK2-overexpressing 293A cells and MDA-MB-231 cells. The harvested cells were lysed in lysis buffer (20 mM Tris pH 7.5, 150 mM NaCl, 1% Triton X-100, 10 mM EDTA, and 10 mM ethylene glycol tetraacetic acid [EGTA]) and immunoprecipitated with anti-Flag antibody (F1804, Sigma-Aldrich, MO, USA) and protein G beads (05 015 952 001, Roche, Basil, Switzerland). VRK2 with attached G beads was used for the kinase assay. After the kinase reaction, proteins were eluted from the beads by denaturation at 95°C.

### Human tissue sample

The study analyzed samples from 20 patients with breast cancer. The patients underwent surgery at Seoul National University Hospital, Seoul, Korea, between 1996 and 1998. This study was reviewed and approved by the Institutional Review Board of Seoul National University Hospital (H-1905-095-1034). This study abides by the principles of the Declaration of Helsinki. All patients signed the informed consent form.

### Cell culture and transfection

MDA-MB-231 cells were grown in Dulbecco’s modified Eagle’s medium supplemented with 10% fetal bovine serum and 100 U/mL each of penicillin and streptomycin. Transient transfections of overexpression vectors and siRNAs were performed using a Microporator (Invitrogen, MA, USA) as described in the manufacturer’s protocols (1680 V, 20 ms, once). According to previous reports, the siRNA sequences were as follows: CGCAGAGUUCCUCACCUGUAdTdT for siADAR1, GCAGUAUGCUCCCGAUCAAdTdT for siApobec3A, and CCUACGCCACCAAUUUCGUdTdT for siCtrl, and CACAAUAGGUUAAUCGAAA for siVRK2 [17–19]. Stable cell lines were generated with pCDNA3-HA-hVRK2-transfected cells. VRK2-expressing cells were selected with 800 µg/mL of G418.

### Colony formation assay

The colony formation assay was performed as described previously [20]. Stable cells generated from MDA-MB-231 were seeded at 2,000 cells per well of a 6-well plate. After 10–12 days of incubation, cells were fixed with 4% paraformaldehyde (PFA) for 30 min at room temperature and were stained with 0.5% crystal violet for 1 h at room temperature. After staining, cells were washed three times with phosphate-buffered saline (PBS). After the cells were completely dried, staining was performed. Colonies were analyzed by measuring the absorbance of crystal violet at 595 nm after its extraction with 20% acetic acid.

### Quantitative PCR

The StepOnePlus Real-time PCR System (Applied Biosystems, MA, USA) was used for mRNA detection and quantification. Quantitative PCR was performed according to the manufacturer’s instructions. The sequences of the forward and reverse primers were as follows: VRK2 (F: TTTAGCATATGATGAAAAGCCAAACTATCA, R: TGAGACTCTTGATATTTCTGTCTTCTCCTT), GAPDH (F: GCCATCAATGACCCCTTCATT, R: GCTCCTGGAAGATGGTGATGG).

### Western blot

Harvested cells and human tissues were lysed with cell lysis buffer (20 mM Tris pH 7.5, 150 mM NaCl, 1% Triton X-100, 10 mM EDTA, and 10 mM EGTA). The total amount of protein was quantified with the Bradford assay. Then, 30 μg of lysates were denatured in a loading buffer (60 mM Tris pH 6.8, 25% glycerol, 2% SDS, 14.4 mM beta-mercaptoethanol, and 0.1% bromophenol blue) and boiled for 5 min at 95°C. SDS polyacrylamide gels were used for all analyses, and the following antibodies were used: VRK2 (sc-365199, Santa Cruz Biotechnology, TX, USA), GAPDH (sc47724, Santa Cruz Biotechnology, TX, USA), HA (A190-108A, Bethyl Laboratories, TX, USA), Dysbindin (ab124967, Abcam, Cambridge, UK), Cyclin D (2978S, Cell Signaling Technology, MA, USA), Ki67 (ab16667, Abcam, Cambridge, UK), Actin (691001, MP Biomedicals, CA, USA), ADAR1 (ab88574, Abcam, Cambridge, UK), Apobec3A (ab38641, Abcam, Cambridge, UK), CREB (9197S, Cell Signaling Technology, MA, USA), Phospho-CREB (9198S, Cell Signaling Technology, MA, USA) and DYKDDDDK Tag (2368S, Cell Signaling Technology, MA, USA).

### Cell Counting Kit-8 (CCK8) assay

MDA-MB-231 stable cells were seeded at 1,000 cells per well of a 96-well plate. After 12, 24, 36, 48, 60, and 72 h of incubation, cells were treated with the CCK-8 solution (kta1020, Abbkine Scientific Co., California, USA) to each well for 1 h in the incubator. After incubation, the absorbance of cells were measured at 450 nm.

### Immunocytochemistry (ICC) and immunofluorescence (IF) staining

To quantify cell proliferation (Ki67), ICC staining was performed on MDA-MB-231 stable cells. Cells were fixed in 4% PFA and washed three times in PBS. Then, cells were incubated with 0.5% Triton X-100 for 30 min at room temperature. Cells were blocked to reduce nonspecific binding using a blocking solution (5% fatal bovine serum+ 2.5% bovine serum albumin + 0.3% Triton X-100 in 10 mL of PBS). After blocking, cells were stained with anti-Ki67 (1:250, ab16667, Abcam, Cambridge, UK) for 2 h at room temperature. Cells were stained with rabbit secondary antibody (1:500, A-11008, Invitrogen, MA, USA) for 2 h at room temperature. After three washes in PBS, cells were stained with Hochest 33342 (1:15000, ab228551, Abcam, Cambridge, UK) for 10 min at room temperature.

To quantify tumor cell growth (Ki67), dysbindin and cyclin D, IF staining was performed on tumor tissues. Tumor tissues were stored in an optimal cutting temperature compound, sectioned in 9 mm, and fixed on the slides. The slides were fixed in 4% PFA and stained with anti-Ki67 (1:250, ab16667, Abcam, Cambridge, UK), anti-dysbindin (1:50, ab124967, Abcam, Cambridge, UK) and anti-cyclin D (1:50, 2978S, Cell Signaling Technology, MA, USA) overnight at 4°C. After three washes in PBS, the slides were stained with rabbit secondary antibody over 2 h at room temperature. Slides were stained with Hochest 33342 for 10 min at room temperature.

The FV3000 Confocal Laser Scanning Microscope (Olympus, Tokyo, Japan) was used for analysis. Fluorescent images were obtained using Coherent® High Performance OBIS™ laser with wavelengths of 405, 488, and 561 nm. z-stack images were also taken using the same imaging system (11–12 stacks, 0.480 μm/slices).

The images were exported using the FV31S-SW program.

### Mouse

All mouse experiments were performed under the guidelines approved by the Institutional Animal Care and Use Committee (POSTECH IACUC protocol code 2022-0072). For *in vivo* experiments, MDA-MB-231 stable cells were implanted subcutaneously in 6-week-old female BALB/c mice after 1 week of acclimation (Orient Bio Inc., Seongnam, South Korea). MDA-MB-231 breast cancer cells were trypsinized, washed in PBS buffer, and injected at 5 × 10^6^ cells per mouse. The tumor volume was measured by caliper and calculated by the formula: Volume = width × length × height × π/6.

### RNA sequencing

In this study, data generated are publicly available in Gene Expression Omnibus at GSE1246468. Normal tissue and breast cancer tissue samples were used for the analysis of VRK2 RNA levels.

### Statistical analysis

All statistical analyses were performed using GraphPad Prism version 9.3.1. The significance of the difference was assessed by the unpaired and paired Student’s *t*-test when comparing two groups. When comparing three groups, the analysis of variance (ANOVA) model was performed with a Tukey adjustment for multiple comparisons. A *p*-value of <0.05 was considered a significant difference. All data are presented as mean ± standard error of the mean (SEM).

## Results

### Sequence variations of human VRK2

**Figure 1:**
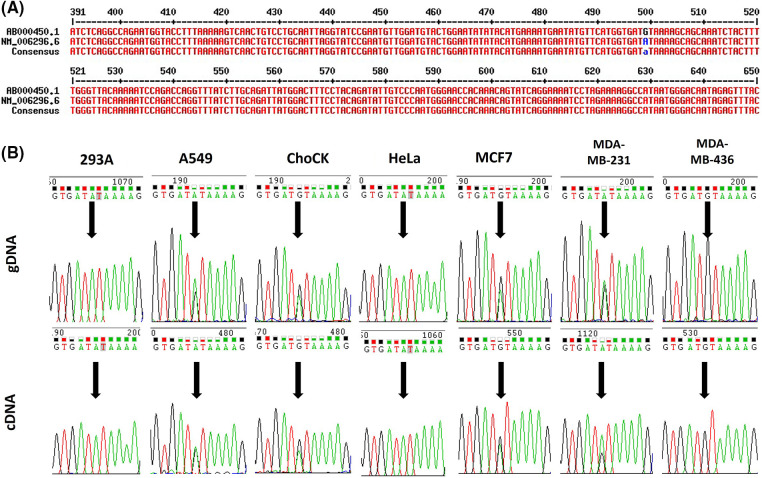
Sequence variations of human VRK2. (A) Alignment of human VRK2 sequences; ab000450.1 and nm_006296.6 (NCBI database) were aligned, and a difference in the sequence at c.499 was detected. (B) Sequencing results of gDNA and cDNA from various cell lines: 293A, A549, ChoCK, HeLa, MCF7, MDA-MB-231 (M231), and MDA-MB-436 (M436). Black arrows indicate the position of c.499.

Various versions of human VRK2 mRNA sequences from the National Center for Biotechnology Information (NCBI) database were aligned, and a base difference at c.499 was identified (Fig. 1A). To determine whether this difference in mRNA sequence was derived from the gDNA, we investigated a reported single nucleotide polymorphism (SNP) of VRK2 from the NCBI database. The c.499 variation was reported as rs1051061. The mRNA sequence variation could have been derived from the gDNA sequence variation, which was reported as the SNP. This variation occurred within the coding sequence; thus, we analyzed whether it was synonymous or nonsynonymous. We found that the A-to-G substitution at c.499 was a missense substitution that changes amino acid 167 from isoleucine (I) to valine (V) within the catalytic motif of VRK2 (HGDIK).

To examine the variation frequency, gDNA and complementary DNA (cDNA) sequences were analyzed from the following cell lines: 293A, A549, HeLa, MCF7, MDA-MB-231, and MDA-MB-436. We found that rs1051061 occurs frequently in these cell lines (Fig. 1B). An RNA–DNA sequence difference (RDD) was also identified in the MDA-MB-231 cell line: the ratio of the A:G variation was 1:1 in the gDNA, whereas it was 2:1 in the cDNA.

### 167I VRK2 has higher kinase activity than 167V VRK2

**Figure 2:**
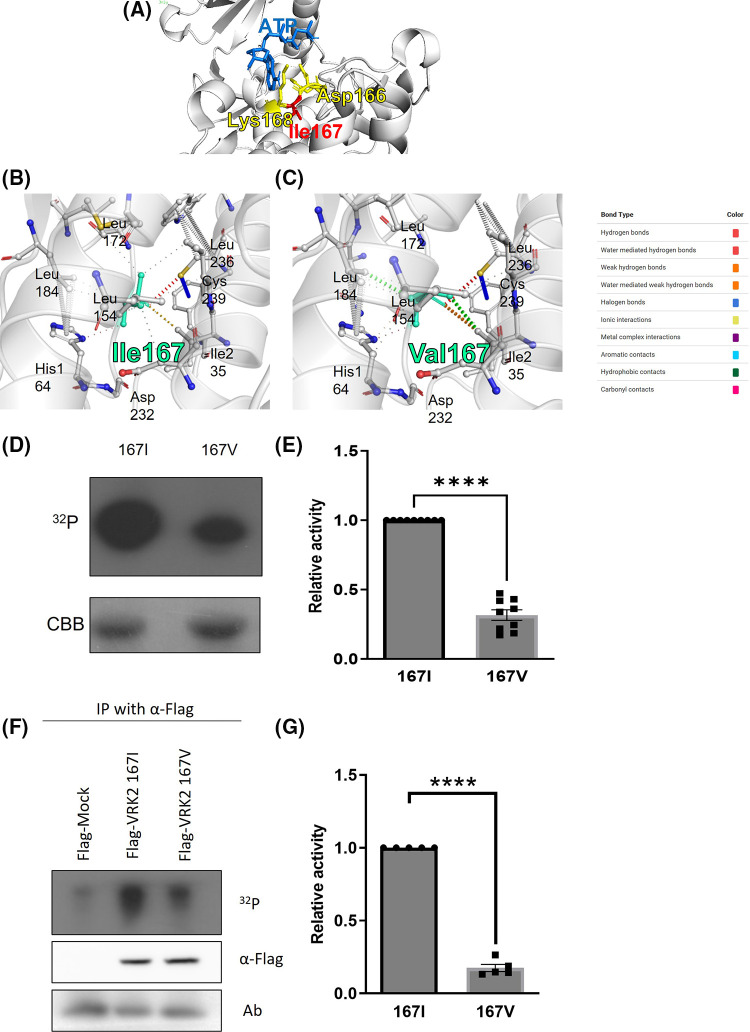
Kinase assays using VRK2 with isoleucine (167I) or valine (167V) substitution. (A) HGDIK is the catalytic motif and ATP binding site. (B) Predicted interatomic interactions of 167I VRK2 using the DynaMut server. The 167I residue appears light green. The line thickness indicates the strength of the interatomic interaction. (C) Predicted interatomic interactions of 167V VRK2 using DynaMut server. The 167V residue appears light green. The line thickness indicates the strength of the interatomic interaction. (D) Results of *in vitro* kinase assays using VRK2 proteins purified from *E. coli*. Each assay used 2 µg of 167V or 167I VRK2. 167I VRK2 displayed higher kinase activity, which was detected by film exposure for radioactivity (^32^P). CBB, Coomassie brilliant blue staining. (E) Quantification analysis of the kinase assay results in (D); *n* = 3. (F) *In vitro* kinase assay using immunoprecipitated VRK2s. The 167I form displayed higher kinase activity than 167V of VRK2. VRK2 amounts were detected by using Western blot. (G) Quantification analysis of the kinase assay results in (F); *n* = 5. All values represent mean ± SEM. ****p < 0.0001, unpaired *t*-test. Each dots in graph represent each samples results of Western blots.

The 167th amino acid residue is within the catalytic motif (HGDIK) of VRK2 [Fig. 2A, predicted structure by PyRx [21]], which is an important site for Adenosine triphosphate (ATP) binding. DynaMut server prediction also suggested that the I167V substitution makes a more rigid protein structure (Figs. 2B and 2C, ΔΔG = 1.097 kcal/mol, stabilizing). The analysis of interatomic interactions showed that the 167V residue had stronger interactions (Fig. 2C, thicker dotted lines) with surrounding residues than that the 167I residue (Fig. 2B), suggesting that protein flexibility is reduced by the amino acid substitution to V [22]. Based on the prediction results, whether amino acid substitution at residue 167 affects VRK2 kinase activity was examined. A kinase assay was performed using radioisotope (P^32^) with *E. coli*-produced and purified 167I and 167V VRK2 proteins. The results indicated that 167I VRK2 had higher kinase activity than 167V VRK2 (Figs. 2D and 2E). This result was confirmed in an *in vitro* kinase assay with 293A cell-synthesized VRK2. Flag-tagged 167I or 167V VRK2 was overexpressed, and VRK2s with anti-Flag antibody were then immunoprecipitated, in which kinase assays were also performed. The results showed that VRK2 produced in 293A cells had the same differences in kinase activities as those observed with proteins purified from transformed *E. coli* (Figs. 2F and 2G).

### VRK2 with higher activity increases cell proliferation

**Figure 3:**
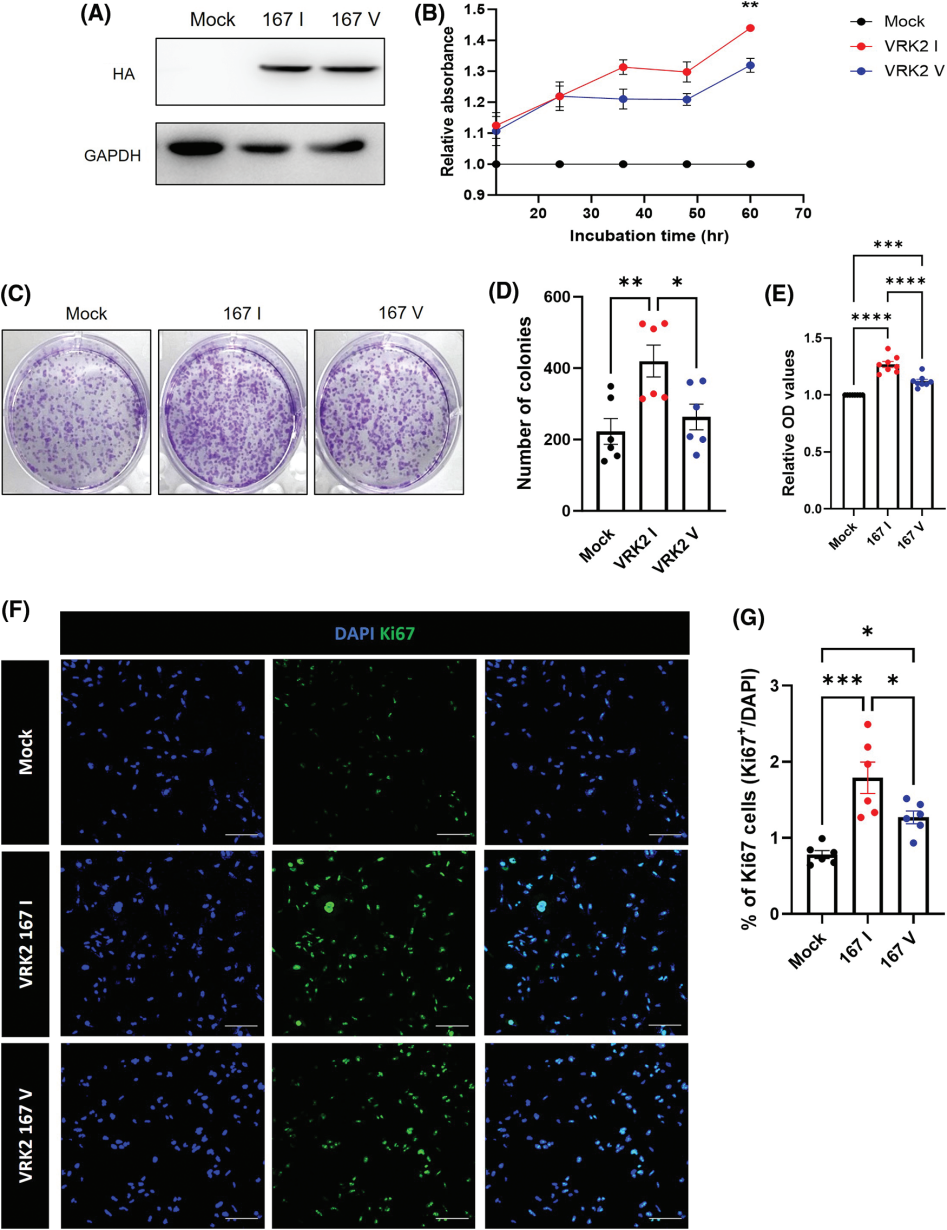
167 I VRK2 increased tumor cell proliferation compared with 167V VRK2. (A) Results of generating stable cells; VRK2 variants overexpressed in MDA-MB-2321 using a pCDNA3-HA vector. (B) CCK-8 assay for measuring cell proliferation. Samples were incubated for 1 h before the absorbance was measured. Representative of *p* values is compared to 167I VRK2 and 167V VRK2 group. (C) Representative images of colony formation assay using stable cell lines. (D) Quantification analysis of the colony numbers results in (C); *n* = 8. (E) The amounts of each colony were measured by their absorbance. Quantification analysis of the colony formation assay results in (C); *n* = 8. (F) Immunocytochemistry (ICC) staining using Ki-67 antibody (green) confirms cell proliferation using MDA-MB-231 stable cell lines. Scale bar = 100 μM. (G) Quantification analysis of the Ki-67 positive cells results in (D); *n* = 6. All values represent mean ± SEM. *p < 0.05, **p < 0.01, ***p < 0.001, ****p < 0.0001, one-way ANOVA and Tukey’s multiple comparison test.

Previous studies showed that VRK2 regulates cancer proliferation [8,9]. Thus, we detected the level of Ki-67, which is a proliferation index, to figure out whether VRK2 affects the cancer proliferation. The results showed that when VRK2 expression was decreased in MDA-MB-231 breast cancer cells, it further reduced the number of Ki-67-positive cells (Suppl. Figs. 1A and 1B). In Fig. 1B, the I form of VRK2 at the 167th amino acid site, which had higher activity, was more frequently found in breast cancer cell. Taken together, we hypothesized that cell proliferation could be altered by VRK2 activity in a kinase activity-dependent manner. To examine the effects of different VRK2 forms on cell proliferation, stable cell lines of MDA-MB-231 that expressed either 167I VRK2 or 167V VRK2 were generated (Fig. 3A). Cell counting kit-8 (CCK-8) assay was performed to determine the effect of 167I VRK2 on cell proliferation. The results showed that 167I VRK2 had higher absorbance than 167V VRK2 (Fig. 3B). Colony formation assay was also performed to confirm the cell proliferation rates of stable cell lines. The results showed increased number of colonies and optical density, which measures the rate of colony formation, at a greater level in the 167I VRK2-expressing cell line than in the 167V VRK2-expressing cell line (Figs. 3C–3E). This observation indicated that 167I VRK2 enhanced the proliferation ability of tumor cells. Then, we analyzed the level of Ki-67 through immunocytochemistry (ICC). As a result, the level of Ki-67 was increased in 167I VRK2-expressing cells than in 167V VRK2-expressing cells (Figs. 3F and 3G). Overall, 167I VRK2 with higher kinase activity enhanced cell proliferation at a greater level than 167V VRK2. These effects on cell proliferation could explain why 167I VRK2 occurs more frequently in cancer tissues than 167V VRK2.

### 167I VRK2 increase cyclin D by reducing dysbindin

In many cancers, especially primary breast tumors, cyclin D expression is increased [23–25]. Therefore, cyclin D induce the tumor growth and progression [26]. Some of studies showed that cyclin D expression is related to cAMP-response element binding protein (CREB)-mediated transcription. Especially, in tumor, CREB affects tumor growth and progression [27,28].

Furthermore, in our previous study, we found that VRK1 regulates cell cycle by phosphorylating CREB at serine 133^th^ site [29]. Therefore, we performed immunoprecipitation analysis to confirm whether VRK2 amino acid substitution affects CREB phosphorylation. However, phospho-CREB level showed no significant difference in mock group and VRK2 variation groups (Suppl. Fig. S2). Our previous study illustrated that VRK2 directly interacted with the dysbindin and decreased its protein stability [30]. A previous study showed that the C-terminal region of dysbindin directly interacts with the C-terminal region of cyclin D, which is regulated by cyclin dependent kinase 4 (CDK4), a cyclin D-binding partner during the cell cycle progression. This interaction leads to the reduction of cyclin D expression and function [31]. So, we tested whether VRK2 regulates dysbindin and cyclin D in breast cancer cells using Western blot analysis. The results showed that MDA-MB-231 cells that were transfected with siVRK2 showed an increase in dysbindin level and a decrease in cyclin D level (Suppl. Figs. S1C–S1F).

**Figure 4:**
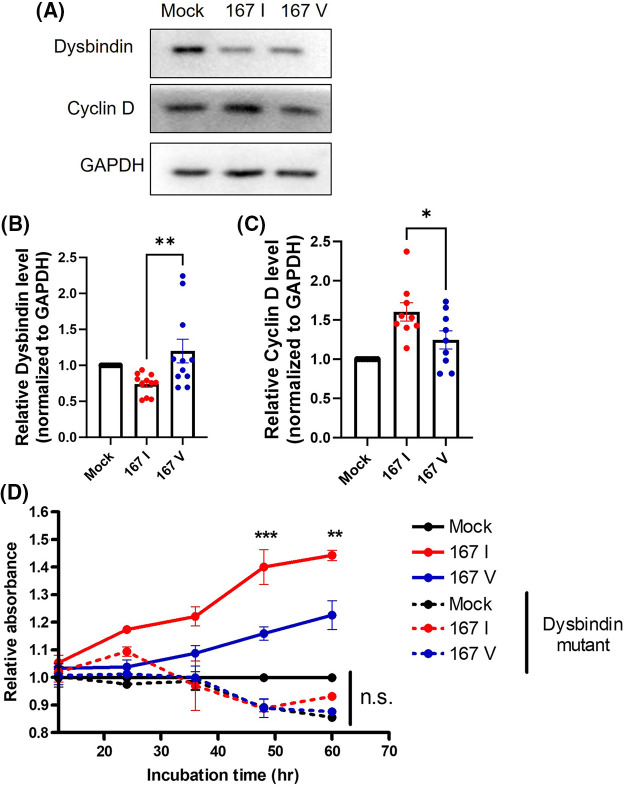
167I VRK2 decreased dysbindin and increased cyclin D expression. (A) Western blot was performed to analyze the expressions of dysbindin and cyclin D in the control, 167I VRK2, and 167V VRK2 cell lines. (B, C) Quantification analysis of the dysbindin and cyclin D expression results in (A); *n* = 11. (D) Dysbindin mutant vector was transfected to stable cells. CCK-8 assay was performed to measure cell proliferation. Samples were incubated for 1 h before the absorbance was measured. Representative of *p* values is compared to 167I VRK2 and 167V VRK2 group. All values represent mean ± SEM. *p < 0.05, **p < 0.01, ***p < 0.001, one-way ANOVA, two-way ANOVA and Tukey’s multiple comparison test. Each dots in graph represent each samples results of Western blots.

According to these results, we hypothesized that 167I VRK2 could increase the cyclin D level by reducing the dysbindin level, increasing cell proliferation. To test this hypothesis, we analyzed the levels of dysbindin and cyclin D through Western blot. The reduction of dysbindin and elevation of cyclin D level was greater in 167I VRK2-expressing cells than in 167V VRK2-expressing cells (Figs. 4A–4C). To confirm whether the tumor proliferation through VRK2 is dependent on dysbindin, we performed the CCK8 assay using the dysbindin mutant vector. In our previous study, we found that VRK2 phosphorylates Ser 297 and Ser 299 of dysbindin [30]. Therefore, we used dysbindin with the mutation at these sites to inhibit its interactions with VRK2, further dysregulating cyclin D. The data showed that dysbindin mutant groups did not show an increase in the proliferation although VRK2 was over-expressed (Fig. 4D).

Taken together, these data indicate that the VRK2-dependent regulation of dysbindin and cyclin D may affect tumor cell proliferation.

### 167I VRK2 increase tumor growth more than 167V in vivo

**Figure 5:**
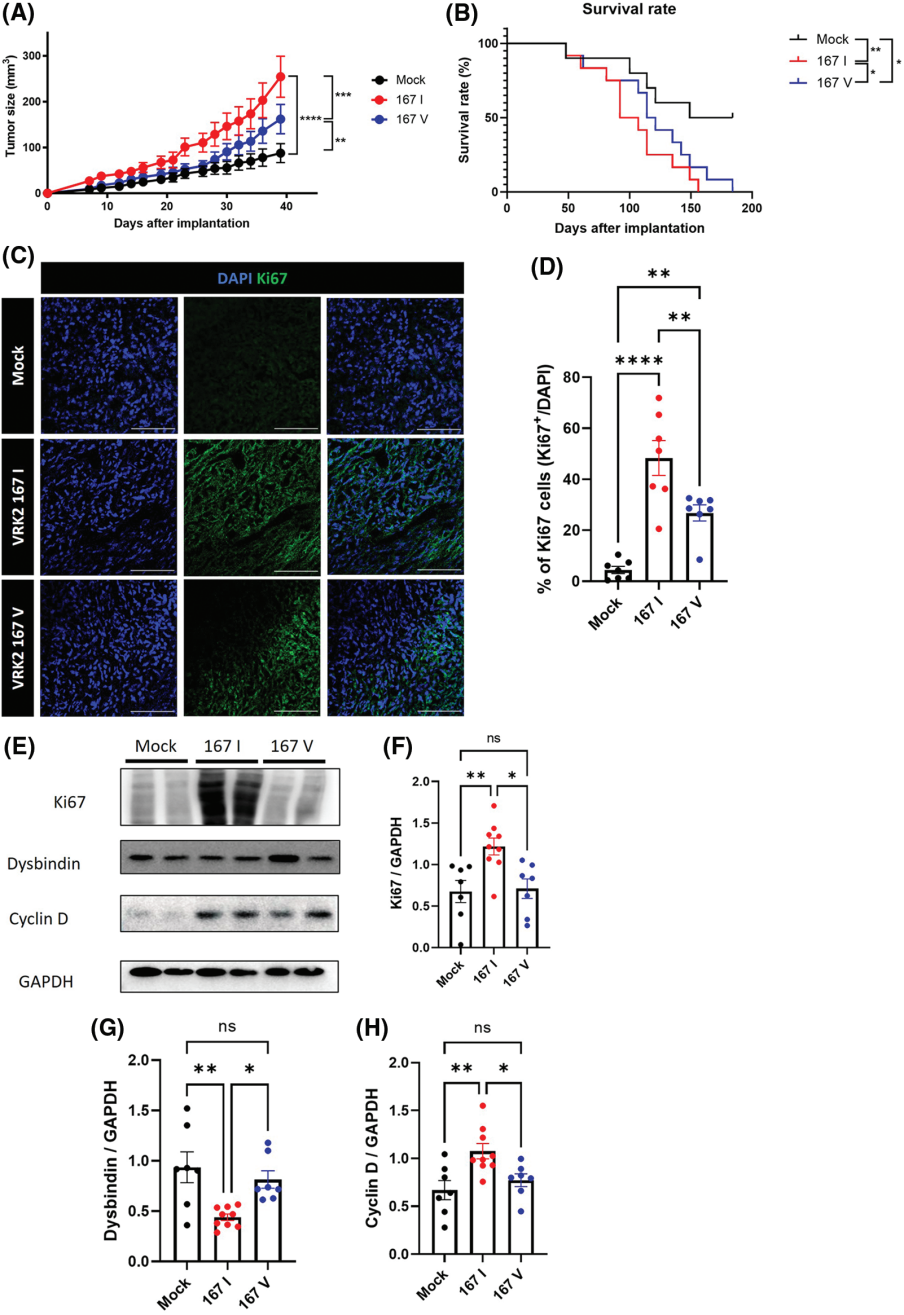
Tumor growth and survival rate were differentially regulated by VRK2 variants *in vivo*. (A, B) MDA-MB-231 stable cell lines were subcutaneously implanted in mice. (A) Tumor growth was measured every 2–3 days using a caliper. (B) Survival rates in the control, 167 I VRK2, and 167 V VRK2 groups. The 167 V VRK2 group showed prolonged survival compared with the 167 I VRK2 group. (C) Representative immunofluorescence (IF) staining data using Ki-67 antibody (green) in tumor tissues. Scale bar = 100 μM. (D) Quantification analysis of Ki-67-positive cell results in (C); *n* = 7 (E). Representative Western blot data of the expressions of Ki-67, dysbindin, and cyclin D in tumor tissues. (F–H) Quantification analysis of Ki-67, dysbindin, and cyclin D expression results in (E); *n* = 8 (control), 10 (167 I VRK2), and 7 (167 V VRK2). All values represent mean ± SEM. *p < 0.05, **p < 0.01, ***p < 0.001, ****p < 0.0001, one-way ANOVA, Log-Rank (Mantel-Cox) test and Tukey’s multiple comparison test. Each dots in graph represent each samples results of Western blots.

Some studies have demonstrated that tumors have high VRK2 expression [8,9]. However, how VRK2 participates in tumor growth is unknown. We previously suggested that 167I VRK2 increases tumor cell proliferation. To further investigate whether different VRK2 variants affect tumor growth *in vivo*, 167I or 167V VRK2-expressing MDA-MB-231 stable cell lines were subcutaneously implanted in BALB/c mice. Tumor growth was measured three times a week. Consequently, both the 167I VRK2 and 167V VRK2 groups had faster tumor growth than the control group. Furthermore, the 167V VRK2 group showed delayed tumor growth when compared with the 167I VRK2 group (Fig. 5A). In addition, 167I VRK2 and 167V VRK2 groups showed shortened survival compared with the control group. Moreover, the 167V VRK2 group showed a higher survival rate than the 167I VRK2 group (Fig. 5B). Collectively, the results confirmed that different amino acid forms of VRK2 were associated with tumor growth and survival rate. Immunofluorescence (IF) staining using Ki-67 antibody was also performed in tumor tissues. The results indicated that 167I VRK2 tumor tissues had a greater number of Ki-67-positive cells than 167V VRK2 tumor tissues (Figs. 5C and 5D). In addition, Western blot and IF staining demonstrated that VRK2 variation affects tumor growth by regulating cyclin D through dysbindin expression (Figs. 5E–5H, Suppl. Fig. S3).

### RNA editing of human breast cancer tissue

**Figure 6:**
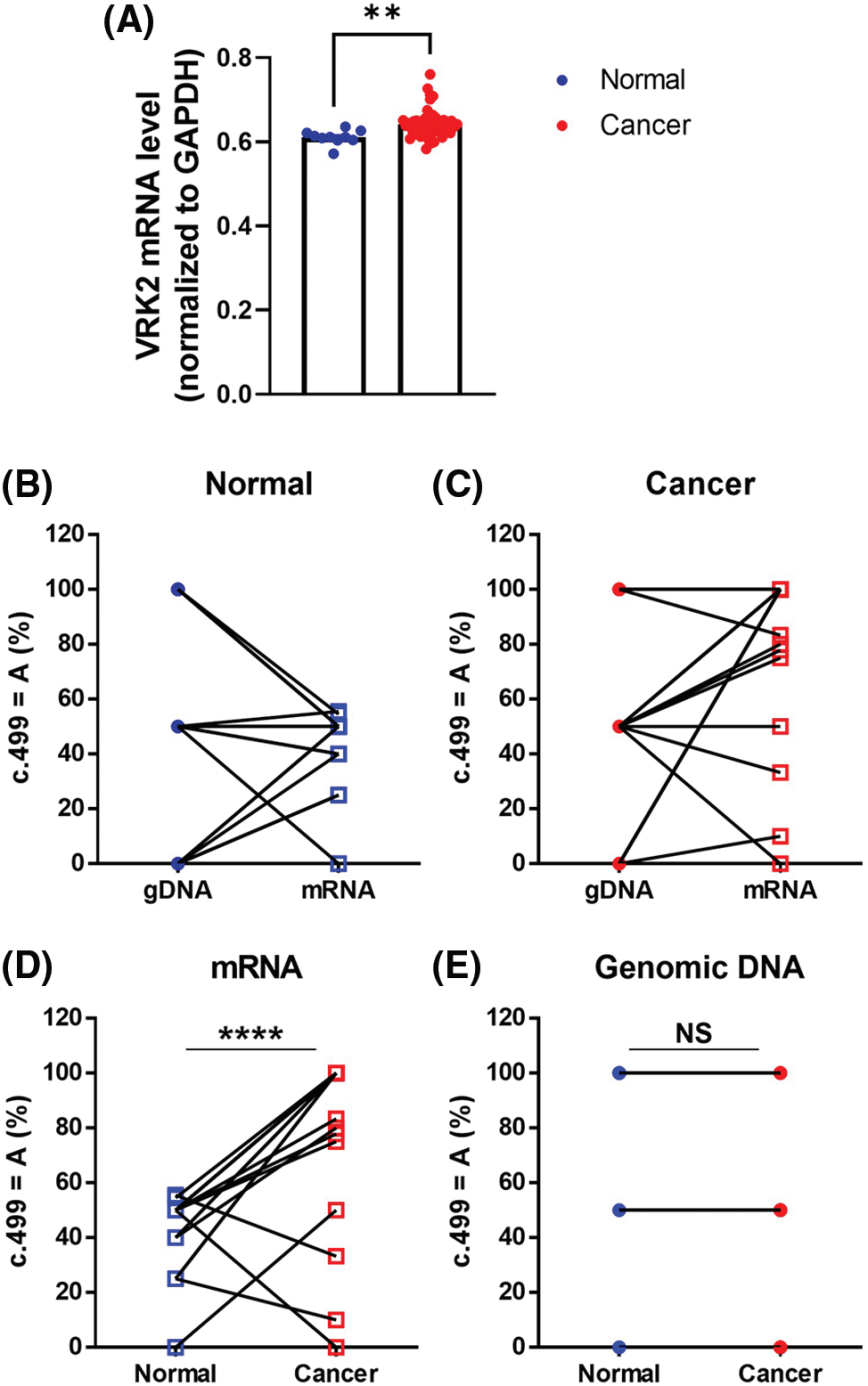
Sequence comparisons of gDNA and mRNA isolated from human breast cancer tissue and adjacent normal tissue samples. (A) RNA sequencing analysis of VRK2 RNA from normal and cancer tissues. (B, C) Proportion of c.499 = A in gDNA and mRNA isolated from normal adjacent tissue (B) and cancer tissue (C). Both A-to-G and G-to-A RNA editing were detected. (D) Proportion of c.499 = A in mRNA isolated from normal adjacent tissues and cancer tissues. The c.499 = A form increased in cancer tissues; *n* = 20, ****p < 0.0001, paired *t*-test. (E) Proportion of c.499 = A in gDNA isolated from normal and cancer tissues. The gDNA sequences did not differ in normal tissue and cancer tissue. All values represent mean ± SEM. **p < 0.01, unpaired *t*-test.

The sequencing analysis results of various cell lines indicated that DNA sequences differ from RNA sequences in the MDA-MB-231 breast cancer cell line (Fig. 1B). To determine whether these differences are also observed in human tissues, RNA sequencing was performed using human breast cancer tissues, and they were compared with those of normal tissues. The RNA level of VRK2 was higher in cancer tissues than in normal tissues (Fig. 6A). We analyzed gDNA and cDNA sequences in human breast cancer tissue samples. The results showed that RDDs were frequently detected (13/20, 65%) in normal breast tissue samples (Fig. 6B). In some patients, the frequency of c.499 = A was higher in cDNA than in gDNA. Other patients displayed a lower frequency of c.499 = A in cDNA than in gDNA. Moreover, the base which does not exist in gDNA, was detected in mRNA in some patients. These results suggest that both A-to-G and G-to-A RNA editing occur at c.499 in the mRNA of VRK2. RDD was observed slightly more frequently in cancer tissues than in normal adjacent tissues (16/20, 80%) (Fig. 6C). Therefore, we compared sequence differences between VRK2 mRNA isolated from normal tissue and cancer tissue. The results showed that the frequency of c.499 = A, which leads to translation of the more active 167I VRK2, was higher in cancer tissue (Fig. 6D). This means that G-to-A editing increases in cancer tissues compared with normal adjacent tissues. However, these differences in VRK2 sequences in normal and cancer tissue were not due to changes in the genomic DNA sequence (Fig. 6E). These combined results indicate that both forms of RNA editing can occur at c.499, and the proportion of c.499 = A increased in the mRNAs of cancer tissues without concomitant change in the DNA sequence. In conclusion, the proportion of c.499 = A in the mRNA sequence of VRK2 may be utilized as the new tumor growth index.

### ADAR1 mediates A-to-G RNA editing

To determine which enzyme mediates RNA editing at c.499 that leads to VRK2 amino acid variation, we overexpressed Flag-tagged ADAR1, which induces A-to-G RNA editing [11,12], in the MDA-MB-231 cell line. ADAR1 overexpression increased the frequency of A-to-G RNA editing (Suppl. Figs. S4A–S4D). This result was confirmed by siRNA knockdown. siADAR1-transfected cells had lower frequency of A-to-G RNA editing (Suppl. Figs. S4E–S4H). Both the overexpression and knockdown experiments indicate that ADAR1 is the enzyme responsible for mediating the A-to-G transition at c.499. On the contrary, the alteration of ADAR1 level did not affect the levels of VRK2 mRNA or protein (Suppl. Figs. S4A, S4B, S4E, and S4F). Although we examined whether the ADAR1 level affects the level of A-to-G editing in human tissue samples, a significant correlation was not observed (Suppl. Fig. S5).

We wanted to further identify the enzyme that mediates G-to-A RNA editing, which occurs more frequently in cancer tissues. One of studies reported an enzyme that caused G-to-A RNA editing, and it is Apobec3A [13]. In the present study, the overexpression and knockdown of Apobec3A were performed; however, the frequency of sequence transition at c.499 was not altered. Therefore, G-to-A RNA editing at this site was not mediated by Apobec3A (Suppl. Fig. S6). G-to-A RNA editing induces the I form of VRK2 at the 167th amino acid site. Therefore, finding the enzyme that targets G-to-A RNA editing may be critical in regulating tumor growth.

## Discussion

Cancer has incidence and mortality rates among diseases; specifically, breast cancer is a fatal disease in women. Thus, researchers continue to study and find the mechanisms related to cancer treatment. For decades, cancer research has focused on finding gDNA mutations in oncogenes or tumor-suppressor genes. In addition to gDNA mutations, RNA editing can create transcriptome diversity that can also contribute to cancer pathogenesis. However, RNA editing alterations cannot be detected by DNA-focused approaches. In this study, we identified a novel RNA editing site in VRK2 that is altered in breast cancer cells and tissues.

RNA editing of VRK2 occurs at sequence c.499, which further changes the amino acid 167 of VRK2. The 167th amino acid is located in the HGDIK catalytic motif of VRK2, where the substitution of 168 lysine (K) with glutamic acid generates an inactive kinase. Therefore, we hypothesized that 167th amino acid substitution may affect VRK2 kinase activity. Indeed, VRK2 kinase activity was changed depending on the 167th amino acid. Interestingly, 167I VRK2 had higher kinase activity than 167V VRK2, even though both I and V have hydrophobic side chains. The amino acid 166 aspartic acid (D) in the catalytic motif of VRK2 interacts with Mg^2+^ ion, which is essential for kinase reaction. During the kinase reaction, the substrate hydroxyl groups, which will be phosphorylated, are oriented toward 166D. The K form at the 168th amino acid site stabilizes the negative charge of the ATP γ-phosphate group [32–34]. DynaMut analysis (http://biosig.unimelb.edu.au/dynamut/) predicted that I167V substitution would induce a conformational change in VRK2 (PDB No. 2v62) [22]. A slight change in the orientation of amino acid side chains was identified in the catalytic motif (Figs. 2A–2C). Moreover, I167V substitution was predicted to make the protein less flexible. The catalytic motif is the site of ATP and substrate binding during the kinase reaction; therefore, the structure should open and close flexibly during catalysis [35]. We can hypothesize that the protein flexibility loss due to I167V substitution may result in reduced kinase activity. Thus, to confirm our hypothesis, further experimental investigation of the 167V VRK2 structure is necessary.

A comparison of VRK2 cDNAs isolated from breast cancer tissues and normal adjacent tissues showed that the frequency of c.499 = A in the mRNA sequence increased in cancer tissues without concomitant changes in the DNA sequence (Fig. 6B). The ratio between A and G at c.499 was approximately 1:1 in VRK2 mRNA isolated from normal tissues, which suggests that A-to-G and G-to-A RNA editing is balanced in normal breast tissues. This may be because both 167I and 167V VRK2s are necessary for normal tissues. By contrast, c.499 = A occurred more frequently in cancer tissues, which indicates that 167I VRK2 is dominant and advantageous for tumor survival. Therefore, we investigated that whether VRK2 variants affect tumor cell proliferation, and the results showed that when compared to 167V VRK2, 167I VRK2 reduced dysbindin and increased cyclin D levels, leading to greater tumor cell proliferation. Due to the regulation of two proteins, the survival rate was also dependent on the VRK2 variant, where 167V VRK2 showed prolonged survival than 167I VRK2. Previously, we demonstrated that VRK2 directly interacted with dysbindin, decreasing its stability [30]. Thus, the greater reduction in dysbindin expression in 167I VRK2 may be due to the stronger interaction of VRK2 and dysbindin than 167V VRK2. Further studies are necessary to confirm this hypothesis by investigating the binding affinity of different VRK2 variants to dysbindin.

Previous studies have reported that a somatic VRK2 mutation was not related to cancer progression [36], and rs1051061 has not been reported as a cancer-related SNP. By contrast, our results indicate that RNA-sequence differences at the rs1051061-corresponding position are associated with breast cancer. Although genetic information is encoded in gDNA, the mRNA template is translated into the functioning protein. RNA editing can alter the mRNA template and further affect the function of expressed proteins. Thus, predicting the tumor growth rate is possible by checking the abundance of various VRK2 transcripts through confirming their RNA sequences at c.499. However, this study could not determine the enzyme that makes c.499 = A. Further investigations are needed to identify the enzyme. In this study, we found that a specific form of 167I VRK2 participates in high tumor proliferation and reduced survival rate. Therefore, finding out the upstream enzyme that makes G-to-A editing at c.499, which changes 167^th^ amino acid from valine to isoleucine, seems to be important. Also, it would be critical to confirm whether inhibiting the activity of the upstream enzyme could actually reduce the change from V form to I form of VRK2, as it could determine the possibility of the enzymes as the anti-cancer therapeutic. Furthermore, we plan to perform gene expression profiles in breast cancer tissues to find out the upregulated genes with G-to-A RNA editing as blocking these upregulated genes could also be beneficial to control tumor proliferation and progression.

## Supplementary Materials

Supplementary Figure S1 Reducing VRK2 expression leads to decreasing cell proliferation through regulating dysbindin and cyclin D expression. MDA-MB-231 cells were transfected using siControl or siVRK2. (A) Representative ICC staining data using Ki-67 antibody (green) in MDA-MB-231 cells. Scale bar = 100 µM. (B) Quantification analysis of Ki-67-positive cell results in (A); n = 5. (C, E) Expression of dysbindin and cyclin D was analyzed by Western blot. (D, F) Quantification analysis graph of the dysbindin and cyclin D expression results in (C, E); n = 4. All values represent mean ± SEM. **p < 0.01, unpaired *t*-test. Each dots in graph represent each samples results of Western blots.

Supplementary Figure S2. Level of CREB in breast cancer cells. MDA-MB-231 cells were transfected using flag-tagged 167I or 167V VRK2 vector. Samples were immunoprecipitated VRK2 using flag antibody. (A) Representative western blot data of CREB level in MDA-MB-231 cells. (B) Quantification analysis graph of the relative phosphor CREB/CREB level results in (A) There’s no significant difference between control group and VRK2 variation groups; *n* = 4. Each dots in graph represent each samples results of Western blots.

Supplementary Figure S3 Area of Dysbindin and Cyclin D in breast cancer tissues. (A) Representative IF data of dysbindin expression (red) in MDA-MB-231 cancer tissues. Scale bar = 100 µM. (B) Quantification analysis graph of the results in (A). Dysbindin area was decreased in VRK2 167 I groups compared to VRK2 167 V groups; *n* = 7. (C) Representative IF images of cyclin D expression (green) in cancer tissues. Scale bar = 100 µM. (D) Quantification analysis of the results in (C). VRK2 167 I groups had more Cyclin D area than 167 V groups; *n* = 7. All values represent mean ± SEM. * p < 0.05, ** p < 0.01, one-way ANOVA test.

Supplementary Figure S4 ADAR1 mediates A-to-G editing at c.499. (A) Representative Western blot data of ADAR1 overexpression in MDA-MB-231 cells. ADAR1 overexpression did not change the VRK2 protein levels. (B) qPCR analysis of VRK2 mRNA level in ADAR1-overexpressing cells. ADAR1 overexpression did not change the VRK2 mRNA level. (C) Representative images of the sequencing analysis of the mRNA from the control (Flag-Mock) and ADAR1-overexpressing (Flag-ADAR1) cells. The level of G increased in ADAR1-overexpressing cells. (D) Quantification analysis of the results in (C). A-to-G RNA editing increased in ADAR1-overexpressing cells; *n* = 6. (E) Representative Western blot data of ADAR1 knockdown in MDA-MB-231 cells. ADAR1 knockdown did not change the VRK2 protein level. (F) qPCR analysis of the VRK2 mRNA level in ADAR1-deficient cells. ADAR1 knockdown did not change the VRK2 mRNA level. (G) Representative images of the sequencing analysis of mRNA from the control siRNA (siCtrl) and siRNA-transfected cells. The level of G decreased in ADAR1-deficient cells. (H) Quantification analysis of the results in (G). A-to-G RNA editing decreased in ADAR1-deficient cells; *n* = 3. All values represent mean ± SEM. * p < 0.05, unpaired *t*-test. Each dots in graph represent each samples results of Western blots.

Supplementary Figure S5 Level of ADAR1 in breast cancer tissues. Graph of the correlation between ADAR1 level and RNA editing level from 20 human breast cancer tissue samples. No correlation was found between the two values. Slope = −0.5295 ± 0.4422, R2 = 0.07376, *p* = 0.2467.

Supplementary Figure S6 Apobec3A does not mediate G-to-A editing at c.499. (A) Representative Western blot data of Apobec3A overexpression in MDA-MB-231 cells. (B) Representative images of the sequencing analysis of mRNA from the control (Flag-Mock) and Apobec3A-overexpressing (Flag-Apobec3A) cells. Apobec3A overexpression did not change the mRNA sequence at c.499. (C) Quantification analysis of the results in (B). A-to-G RNA editing was not changed in Apobec3A-overexpressing cells; *n* = 3. (D) Representative Western blot data of Apobec3A knockdown in MDA-MB-231 cells. (E) Representative images of the sequencing analysis of mRNA from the control siRNA (siCtrl) and siApobec3A-transfected cells. Apobec3A knockdown did not change the mRNA sequence at c.499. (F) Quantification analysis of the results in (E). A-to-G RNA editing was not altered by Apobec3A knockdown; *n* = 3. All values represent mean ± SEM. Each dots in graph represent each samples results of Western blots.

## Data Availability

All the data and materials generated for this study are available on request to the corresponding author.

## Supplementary Materials

The supplementary material is available online at https://doi.org/10.32604/or.2023.031031.
