# Supplementary figures and images for: Vaccinia-related kinase 2 variants differentially affect breast cancer growth by regulating kinase activity
Source: Oncol Res. 2023 Dec 28;32(2):421–32. doi: 10.32604/or.2023.031031 (PMC10765118; doi:10.32604/or.2023.031031)

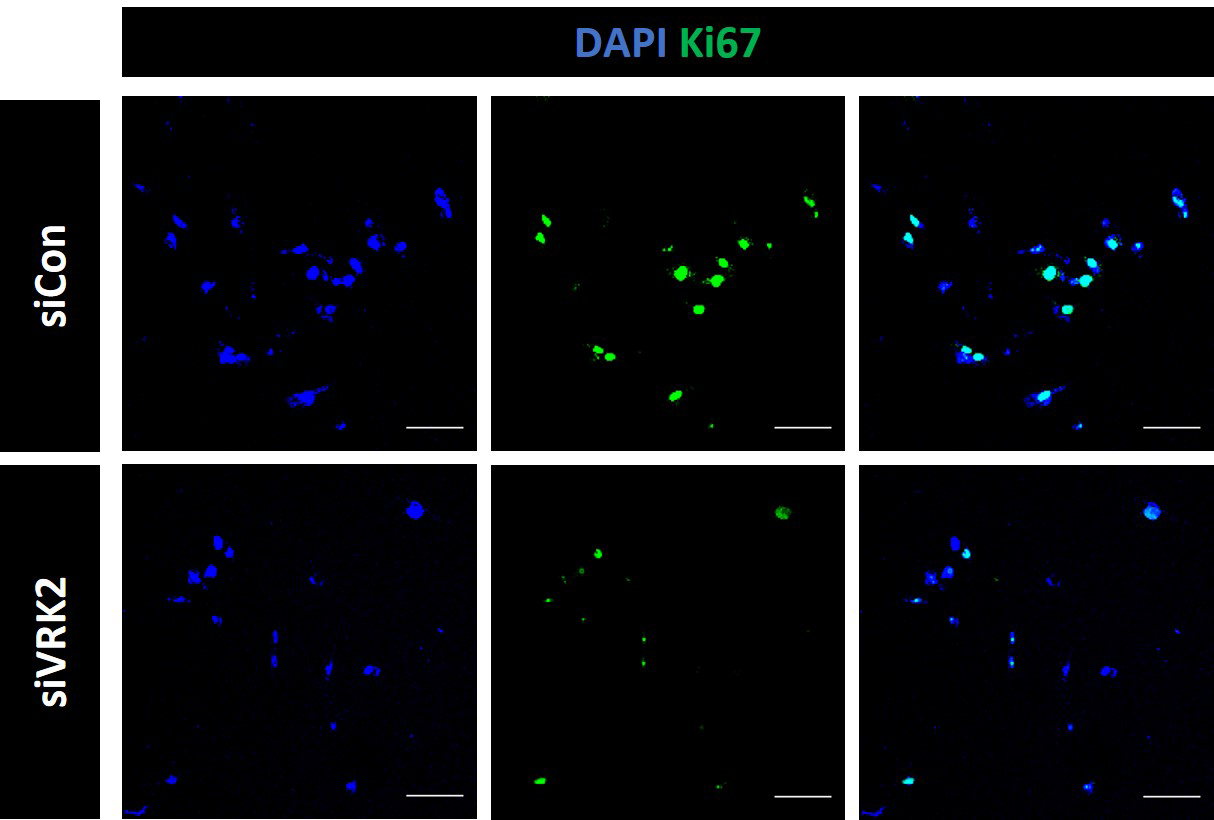

Supplement: Supplementary Figure S1 [file OncolRes-32-31031-s001a.tif]

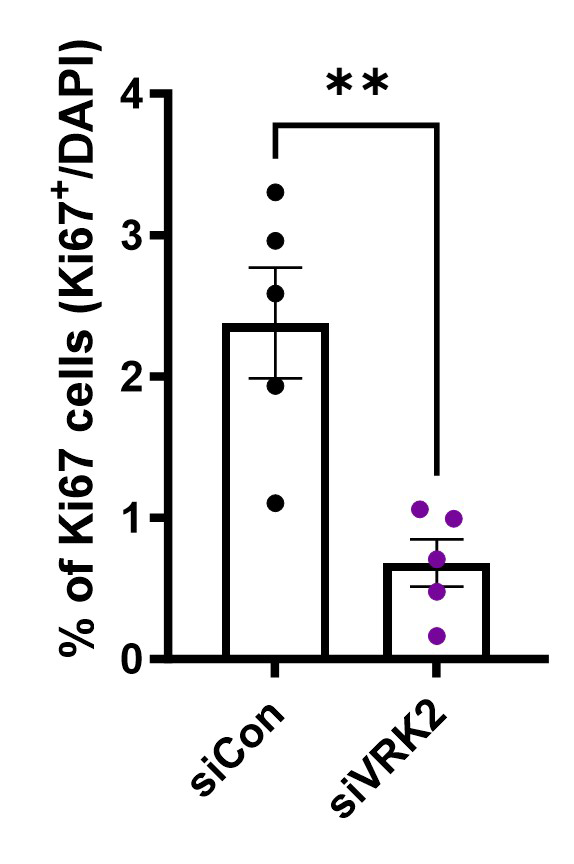

Supplement: Supplementary Figure S1 [file OncolRes-32-31031-s001b.tif]

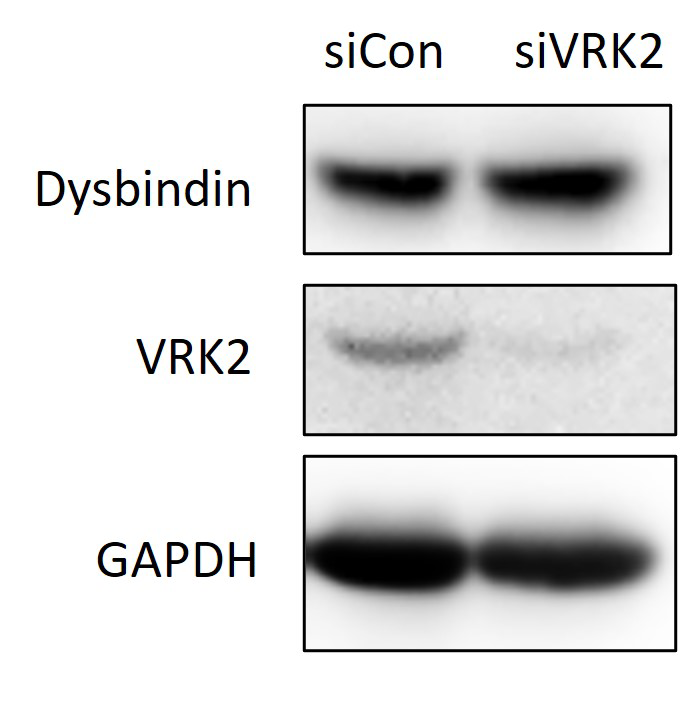

Supplement: Supplementary Figure S1 [file OncolRes-32-31031-s001c.tif]

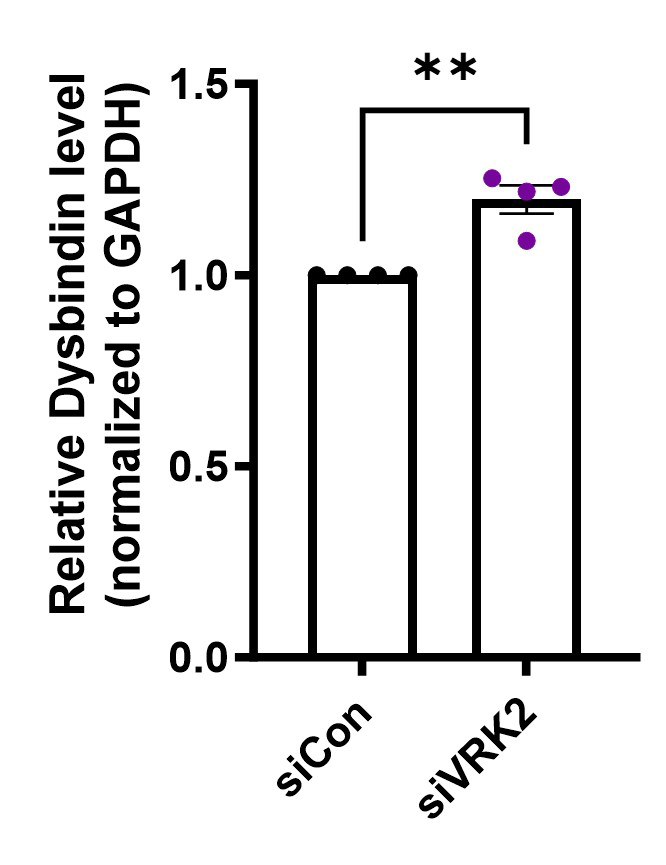

Supplement: Supplementary Figure S1 [file OncolRes-32-31031-s001d.tif]

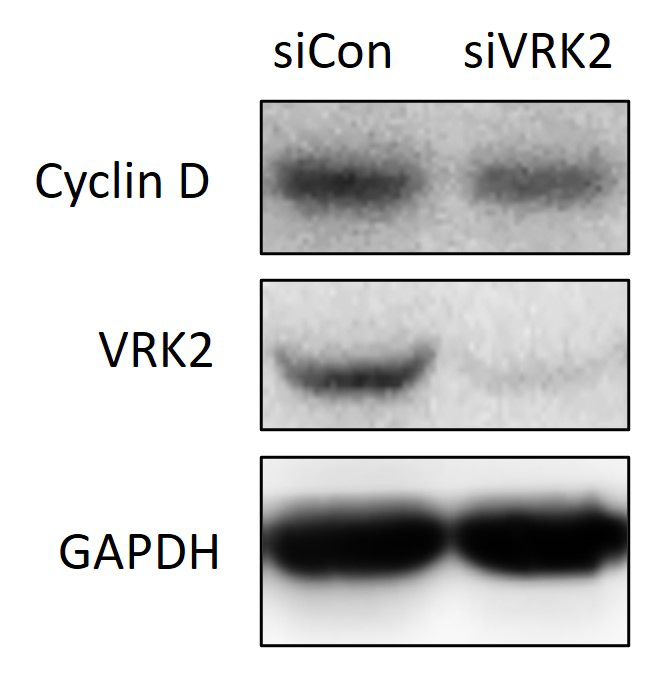

Supplement: Supplementary Figure S1 [file OncolRes-32-31031-s001e.tif]

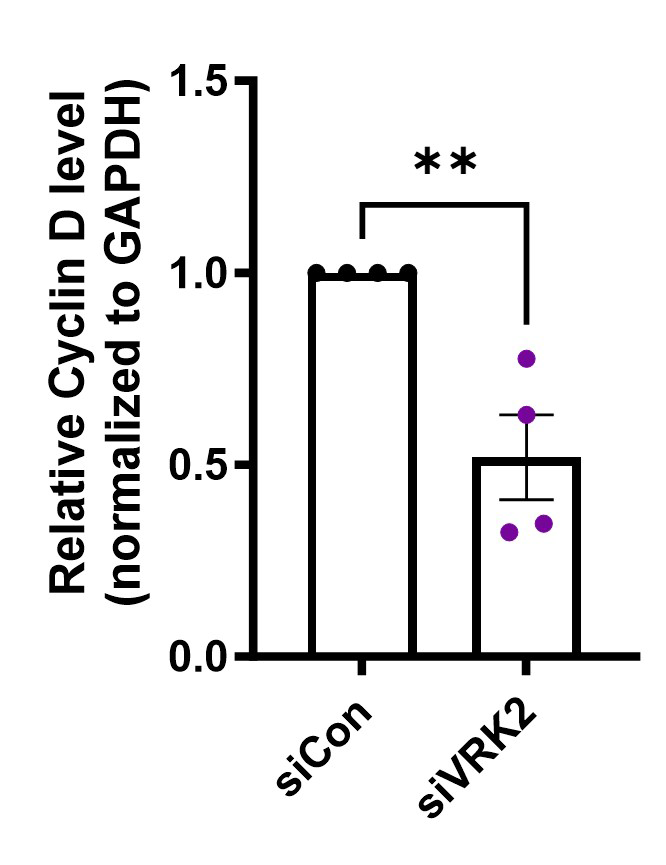

Supplement: Supplementary Figure S1 [file OncolRes-32-31031-s001f.tif]

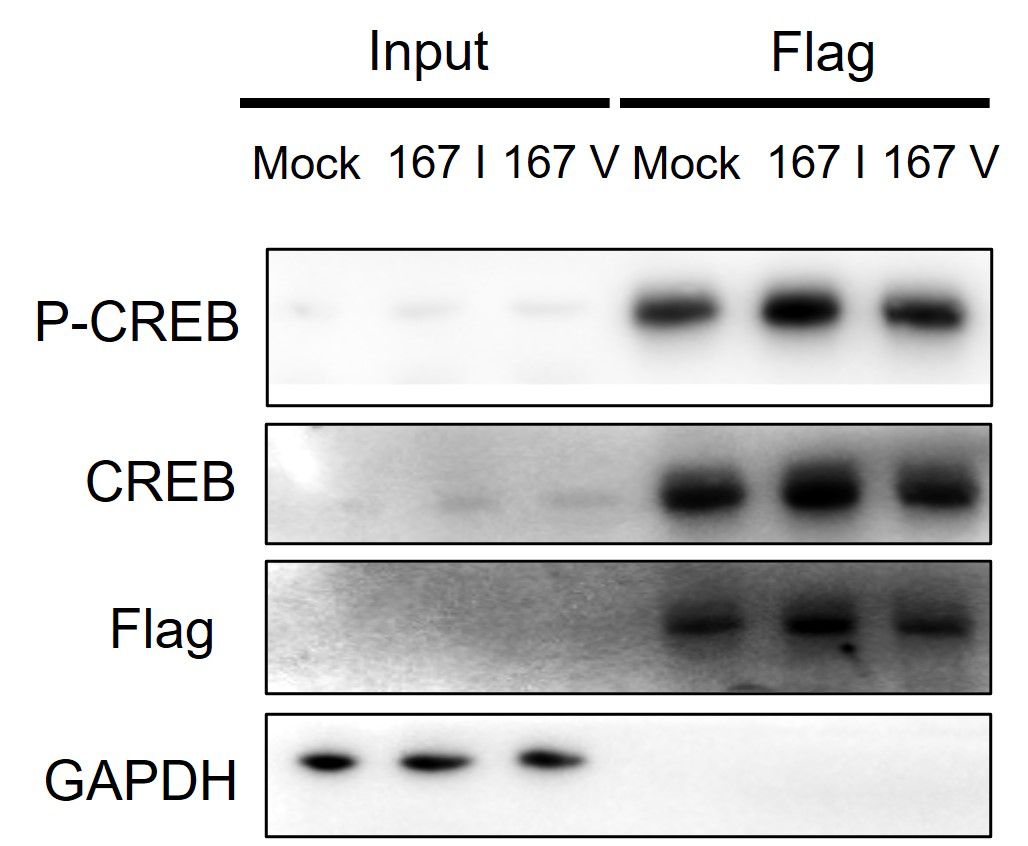

Supplement: Supplementary Figure S2. [file OncolRes-32-31031-s002a.tif]

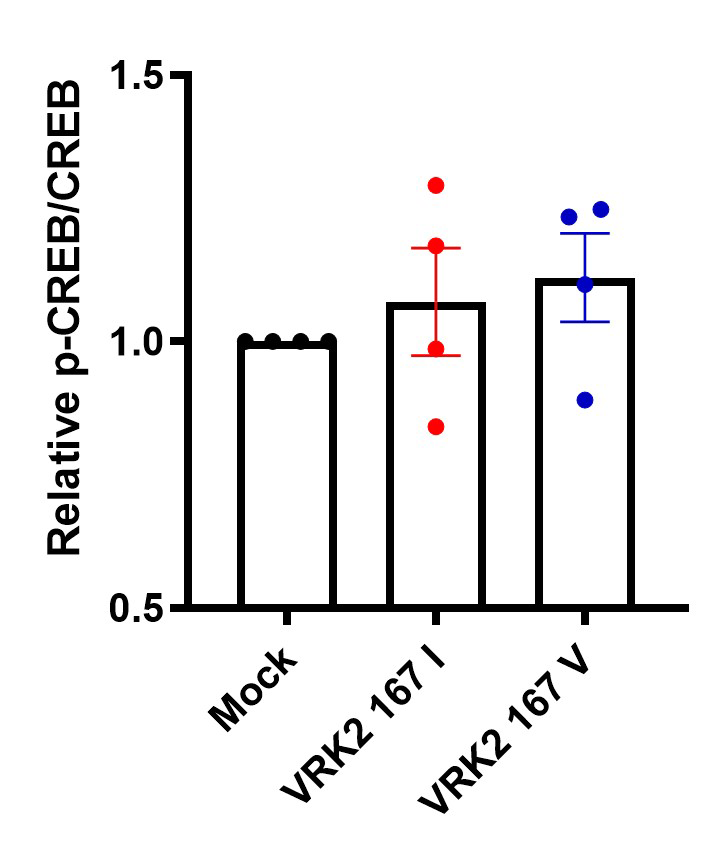

Supplement: Supplementary Figure S2. [file OncolRes-32-31031-s002b.tif]

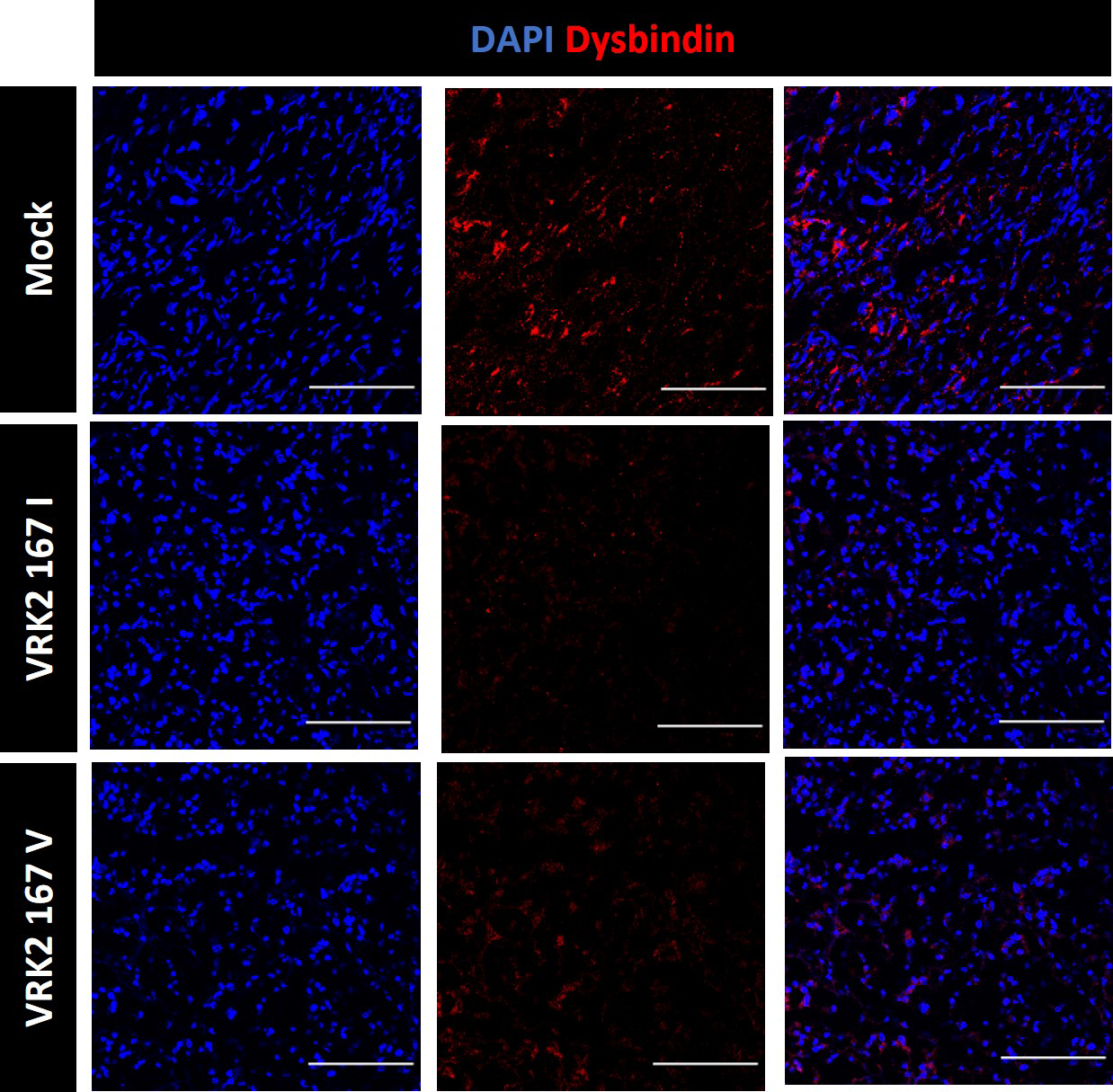

Supplement: Supplementary Figure S3 [file OncolRes-32-31031-s003a.tif]

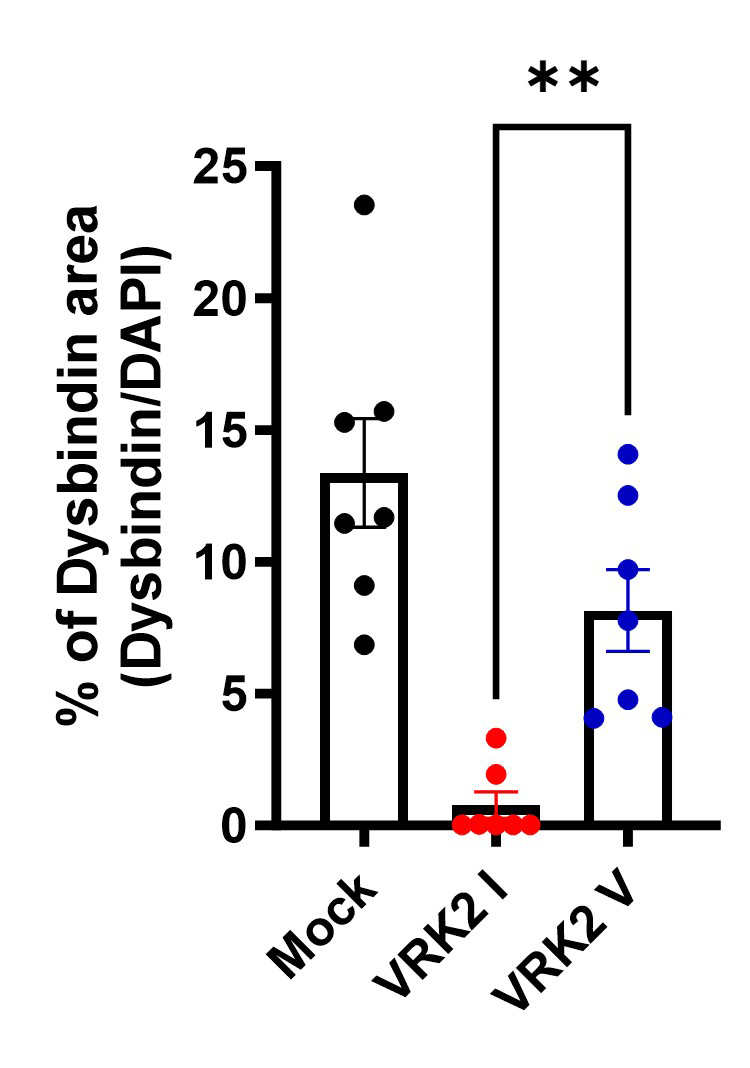

Supplement: Supplementary Figure S3 [file OncolRes-32-31031-s003b.tif]

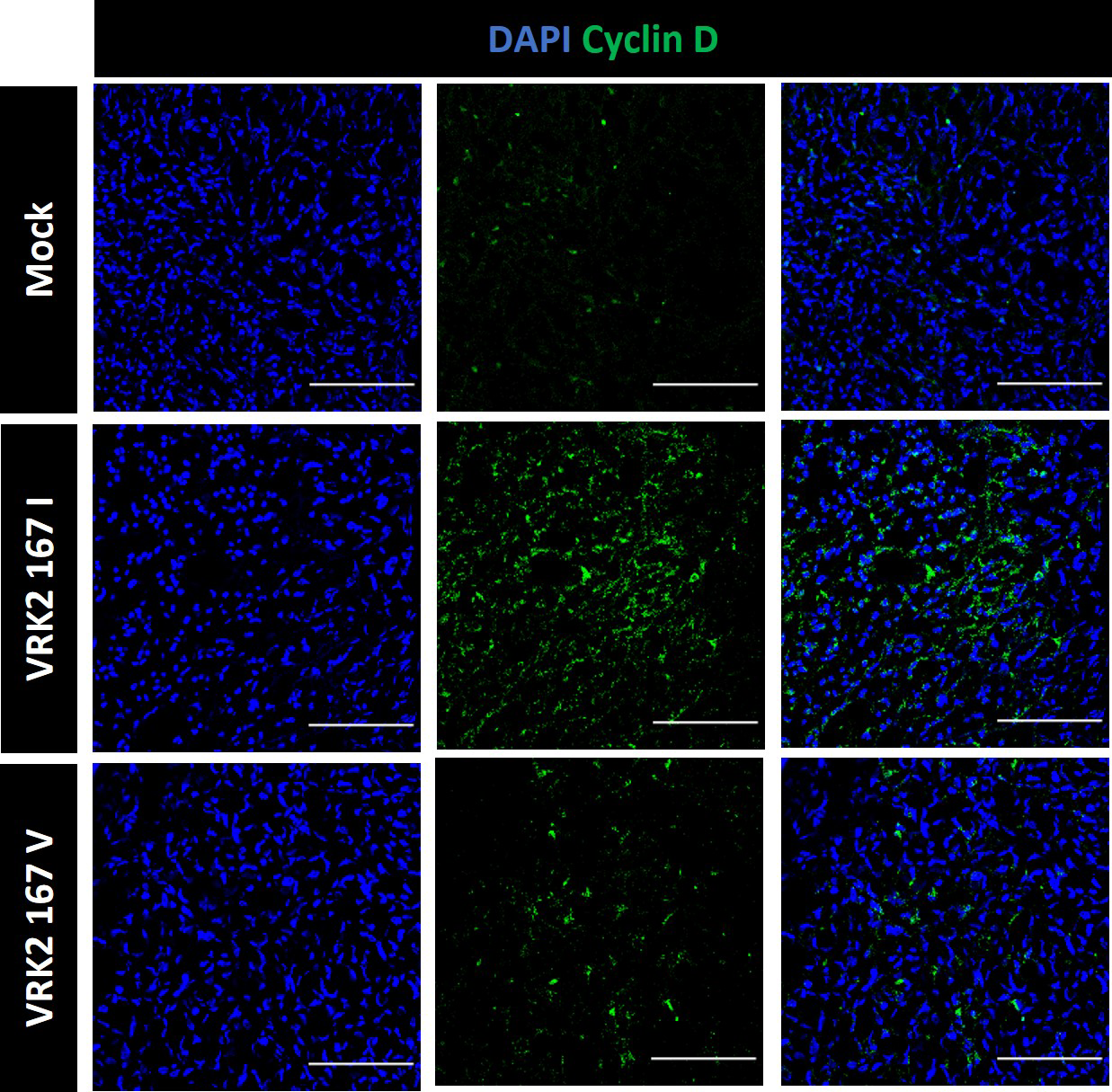

Supplement: Supplementary Figure S3 [file OncolRes-32-31031-s003c.tif]

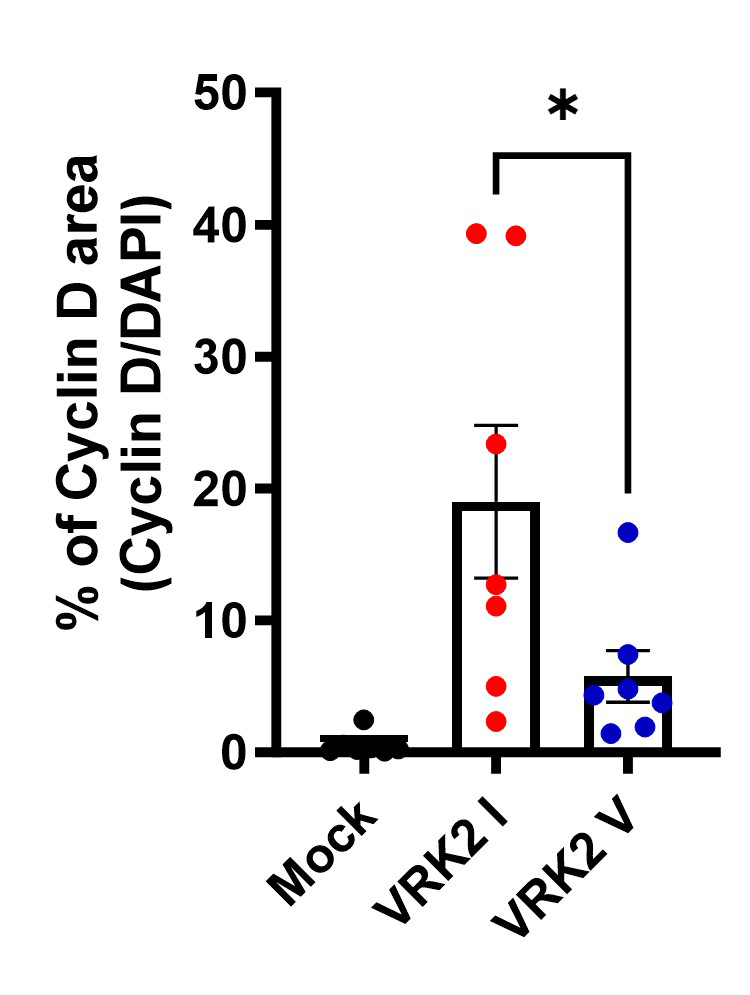

Supplement: Supplementary Figure S3 [file OncolRes-32-31031-s003d.tif]

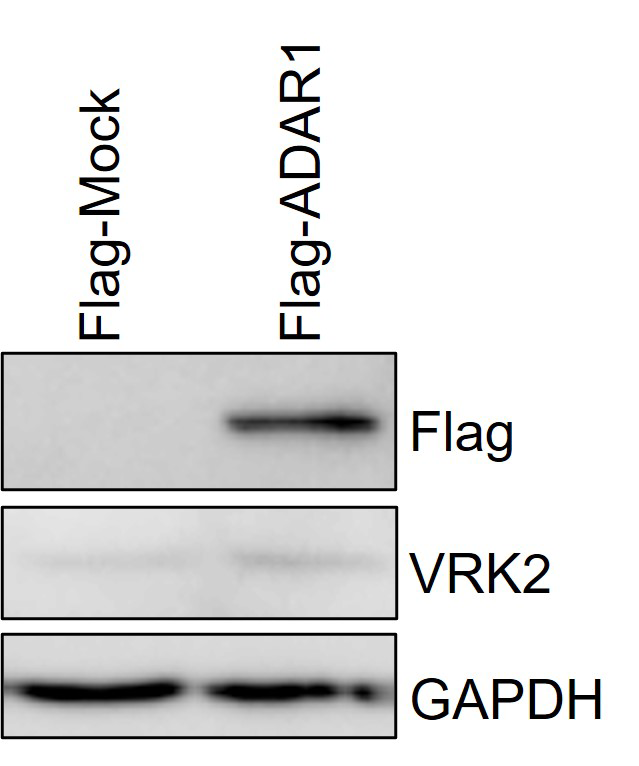

Supplement: Supplementary Figure S4 [file OncolRes-32-31031-s004a.tif]

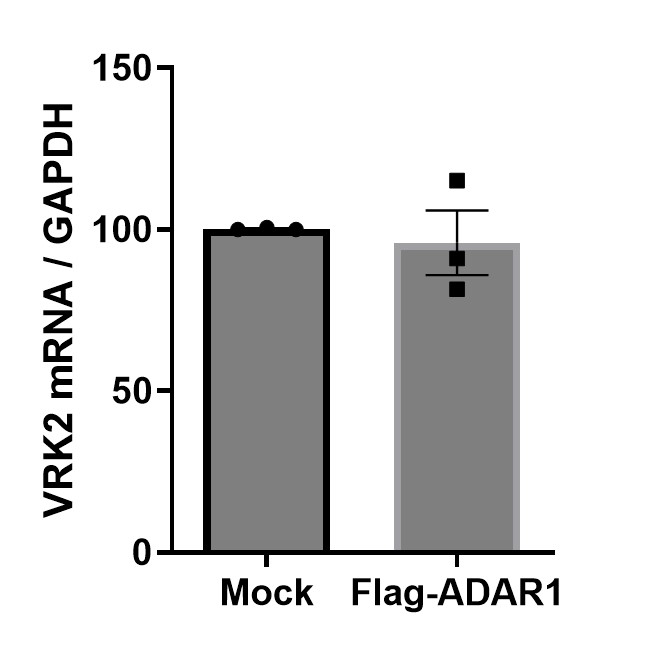

Supplement: Supplementary Figure S4 [file OncolRes-32-31031-s004b.tif]

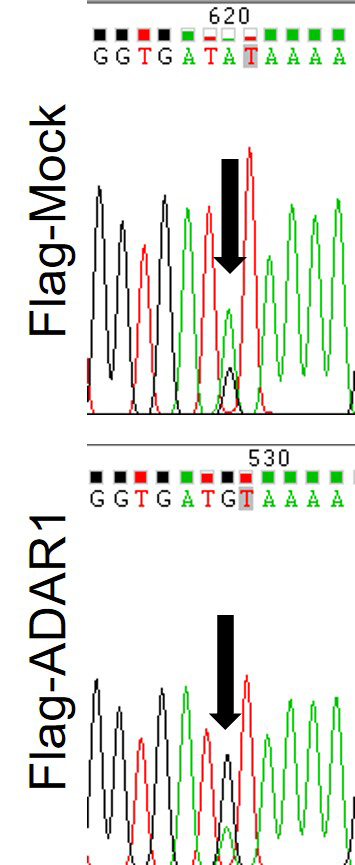

Supplement: Supplementary Figure S4 [file OncolRes-32-31031-s004c.tif]

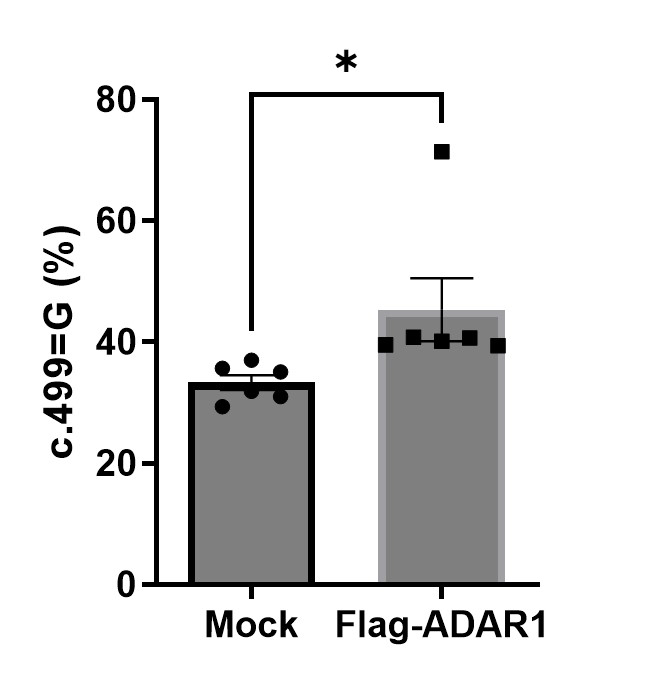

Supplement: Supplementary Figure S4 [file OncolRes-32-31031-s004d.tif]

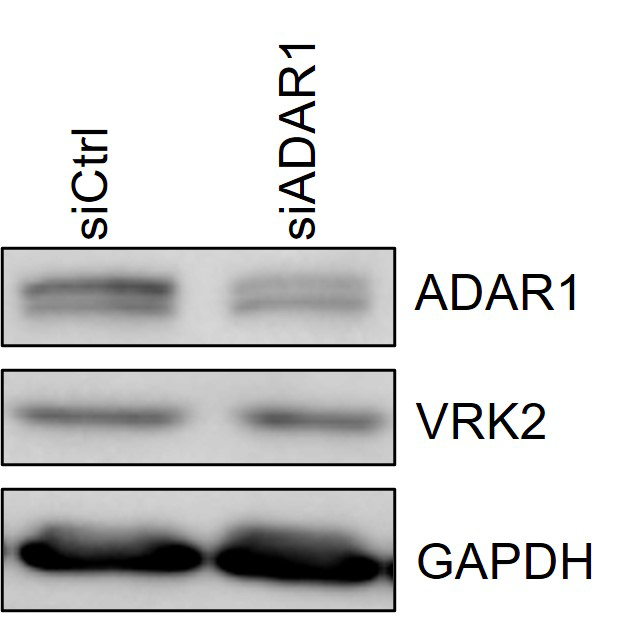

Supplement: Supplementary Figure S4 [file OncolRes-32-31031-s004e.tif]

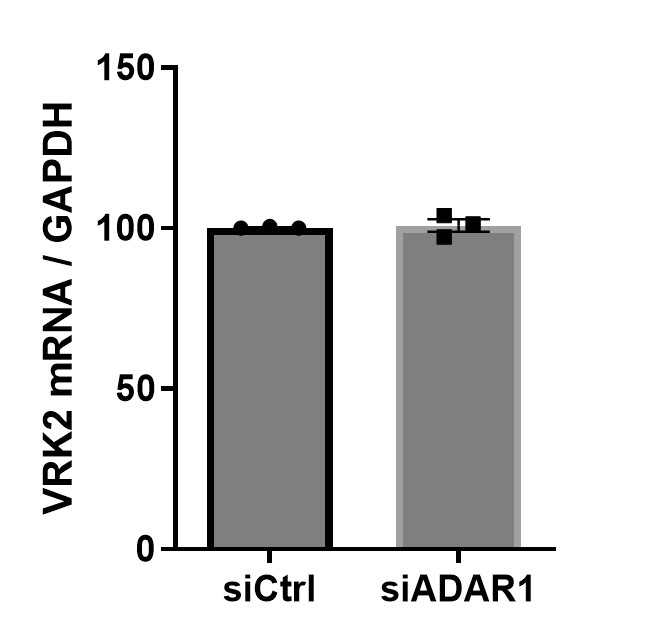

Supplement: Supplementary Figure S4 [file OncolRes-32-31031-s004f.tif]

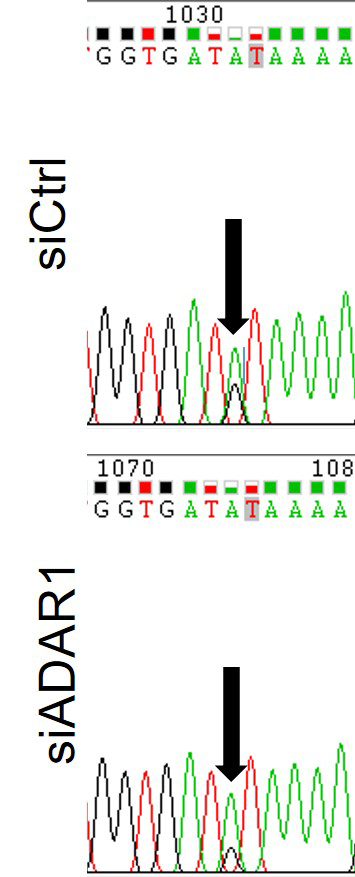

Supplement: Supplementary Figure S4 [file OncolRes-32-31031-s004g.tif]

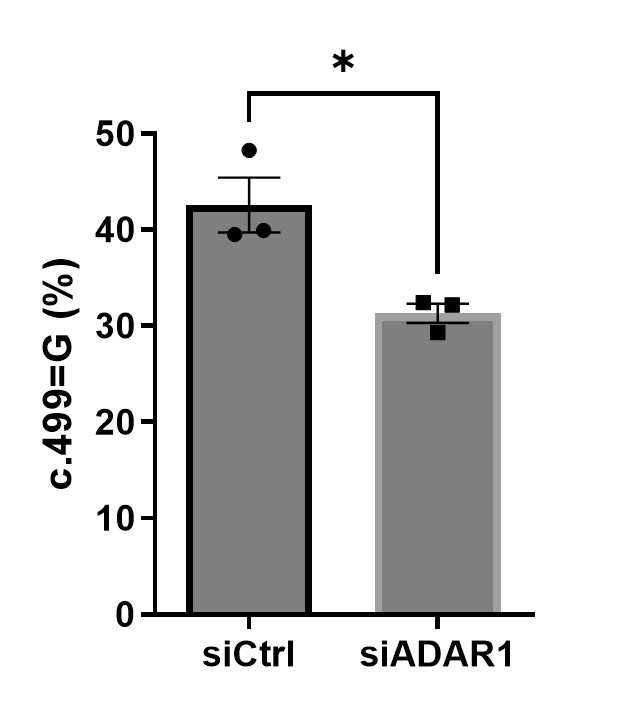

Supplement: Supplementary Figure S4 [file OncolRes-32-31031-s004h.tif]

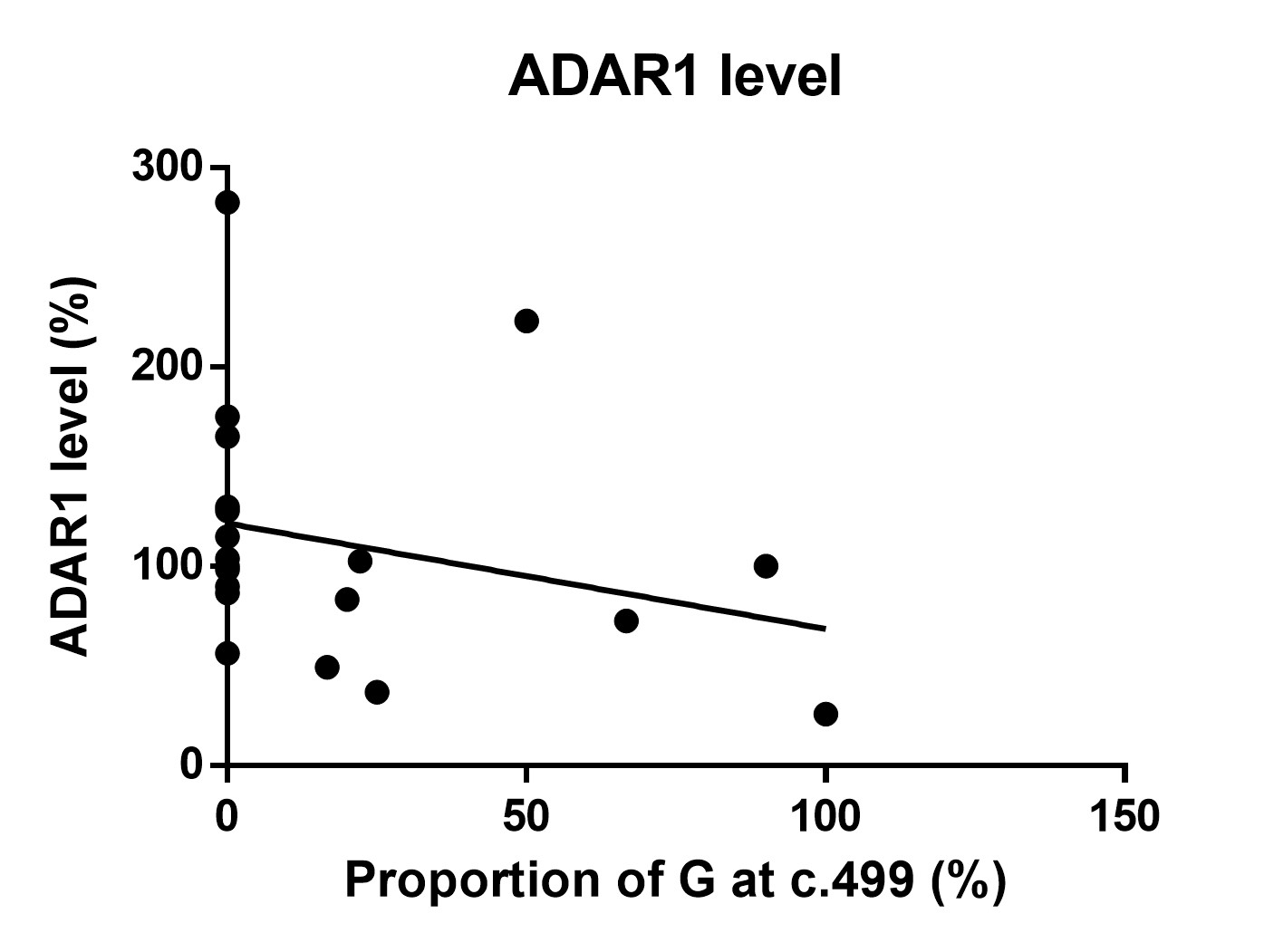

Supplement: Supplementary Figure S5 [file OncolRes-32-31031-s005.tif]

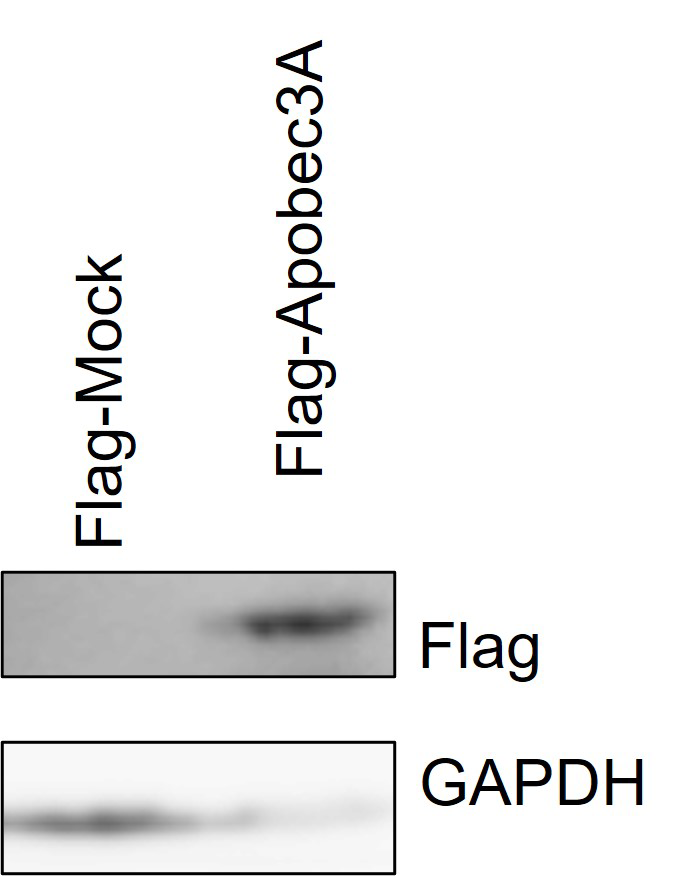

Supplement: Supplementary Figure S6 [file OncolRes-32-31031-s006a.tif]

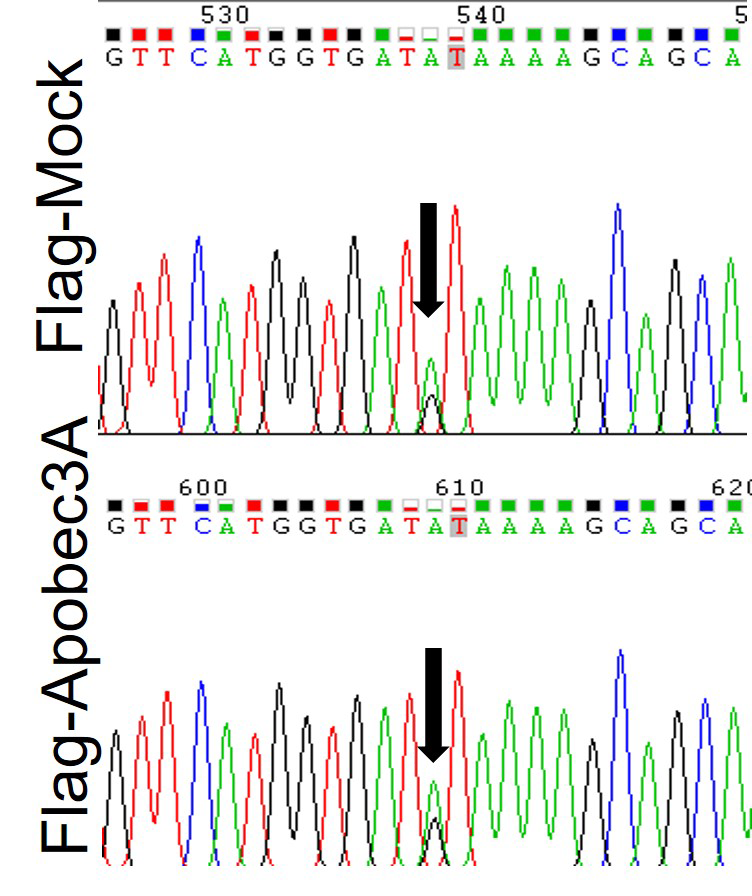

Supplement: Supplementary Figure S6 [file OncolRes-32-31031-s006b.tif]

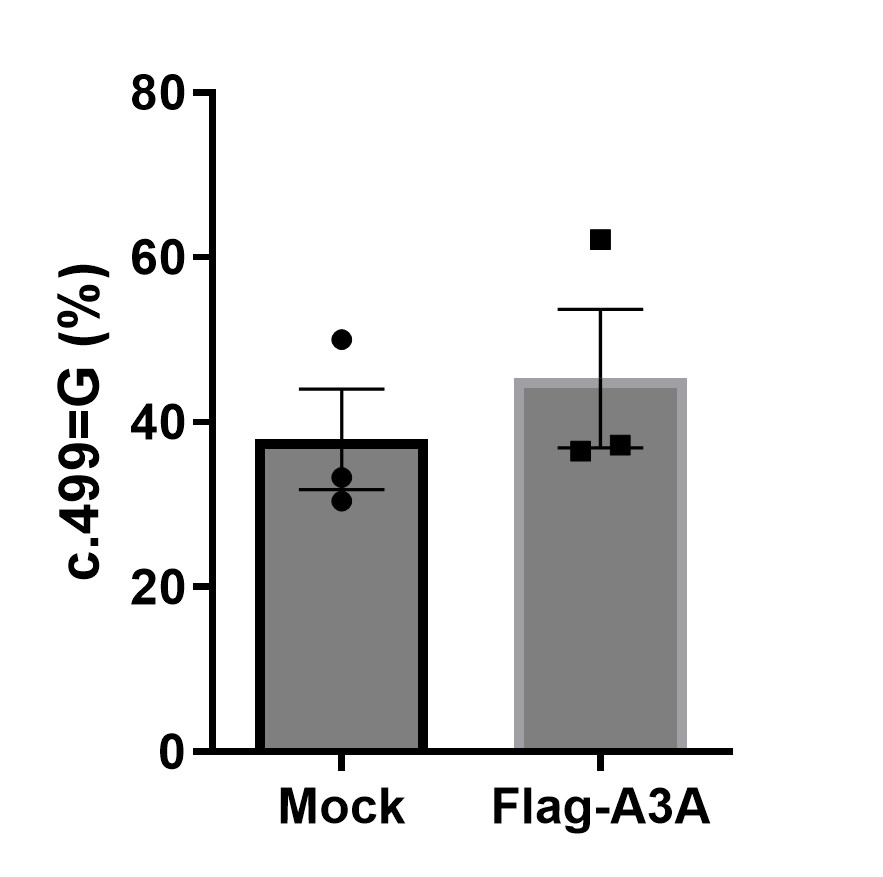

Supplement: Supplementary Figure S6 [file OncolRes-32-31031-s006c.tif]

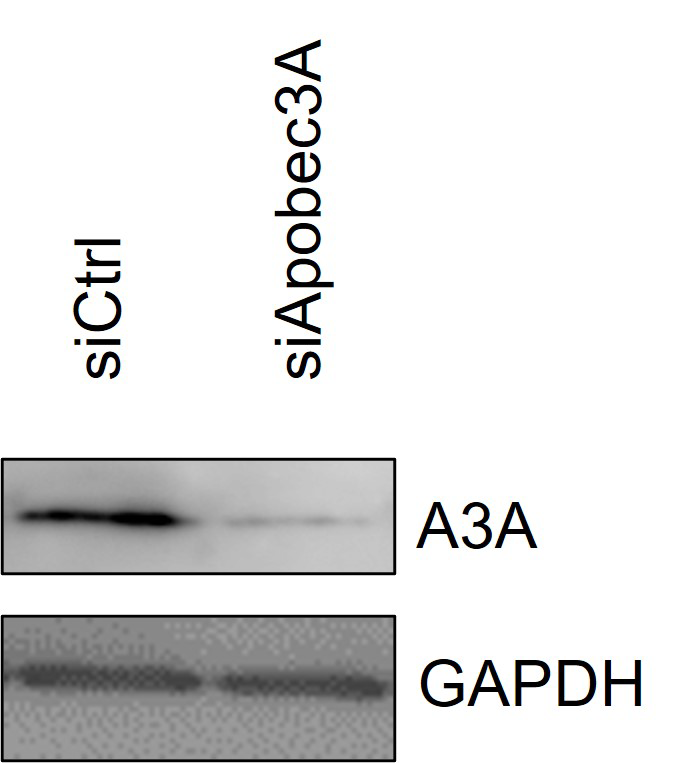

Supplement: Supplementary Figure S6 [file OncolRes-32-31031-s006d.tif]

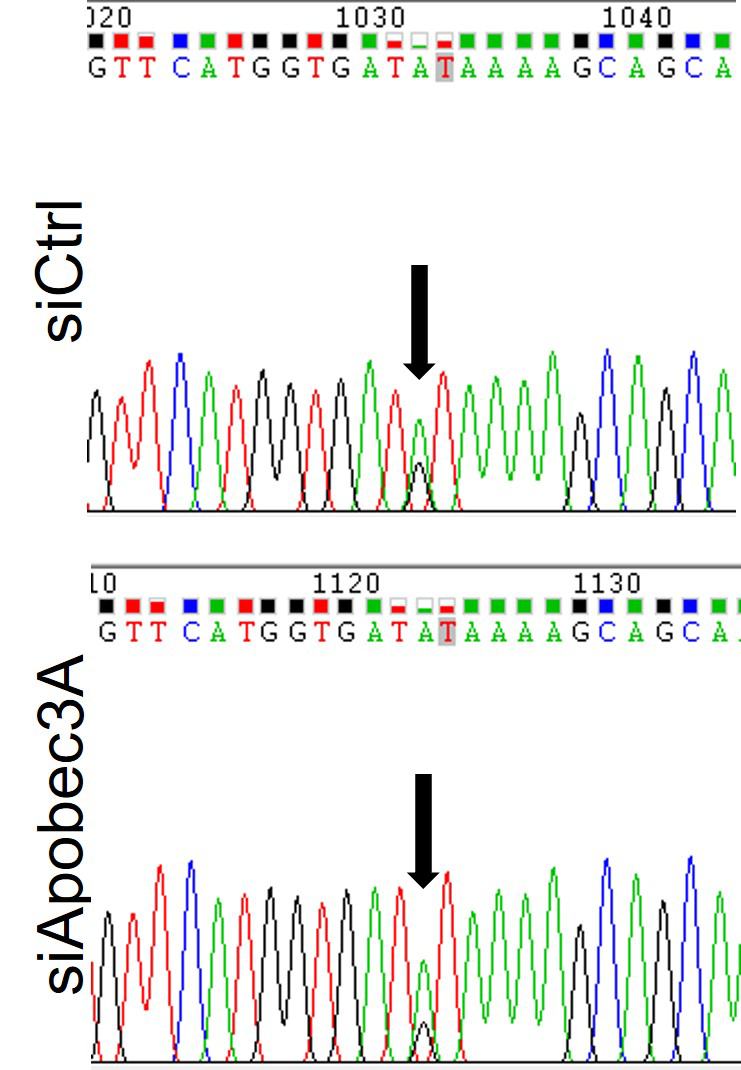

Supplement: Supplementary Figure S6 [file OncolRes-32-31031-s006e.tif]

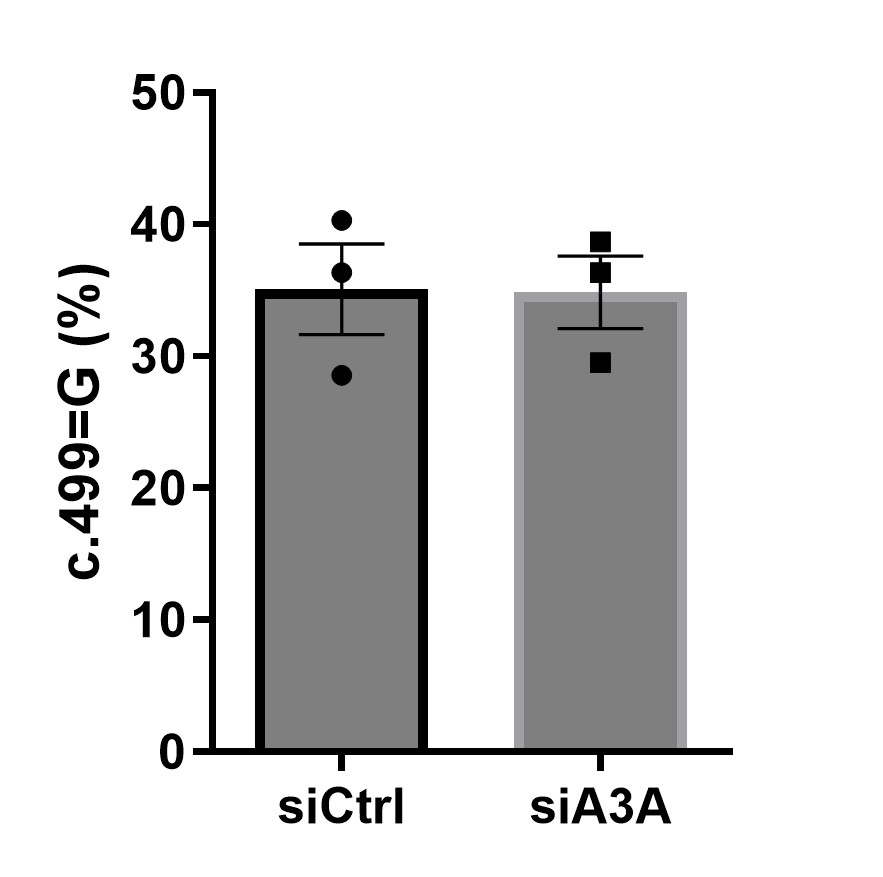

Supplement: Supplementary Figure S6 [file OncolRes-32-31031-s006f.tif]
